# Association of *Mycoplasma canis* with Fertility Disorders in Dogs: A Case Study Supported by Clinical Examination, PCR, 16S Microbiota Profiling, and Serology

**DOI:** 10.3390/pathogens13050391

**Published:** 2024-05-08

**Authors:** Sara Suhadolc Scholten, Brigita Slavec, Primož Klinc, Nataša Tozon, Bojan Papić, Saša Koprivec

**Affiliations:** 1 Small Animal Clinic, Veterinary Faculty, University of Ljubljana, Gerbičeva 60, 1000 Ljubljana, Slovenia; sara.suhadolcscholten@vf.uni-lj.si (S.S.S.); natasa.tozon@vf.uni-lj.si (N.T.); 2 Institute of Poultry, Birds, Small Mammals, and Reptiles, Veterinary Faculty, University of Ljubljana, Gerbičeva 60, 1000 Ljubljana, Slovenia; brigita.slavec@vf.uni-lj.si; 3 Clinic for Reproduction and Large Animals, Veterinary Faculty, University of Ljubljana, Gerbičeva 60, 1000 Ljubljana, Slovenia; 4 Institute of Microbiology and Parasitology, Veterinary Faculty, University of Ljubljana, Gerbičeva 60, 1000 Ljubljana, Slovenia; 5 Institute of Preclinical Sciences, Veterinary Faculty, University of Ljubljana, Gerbičeva 60, 1000 Ljubljana, Slovenia

**Keywords:** *Mycoplasma canis*, canine fertility disorders, vaginal swab, preputial swab, PCR, 16S microbiota profiling, specific antibodies

## Abstract

The role of *Mycoplasma canis* in canine fertility disorders is still poorly understood. As infection is often asymptomatic, there is an increasing need for appropriate diagnostic methods and treatment plans that would allow the reliable detection of *M. canis* infection and rapid alleviation of infection symptoms in affected dogs. In this study, we included 14 dogs with fertility problems and 16 dogs without fertility disorder signs. We compared clinical examination data and selected laboratory parameters (hematology and biochemistry) between the groups. We performed PCR-based detection of *M. canis* and 16S rRNA gene-based microbiota profiling of DNA isolated from vaginal and preputial swabs. Dog sera were tested for the presence of *M. canis*-specific antibodies. Hematological and selected biochemical parameters showed no differences between groups. PCR-based detection of *M. canis* in the samples was consistent with the results of 16S microbiota profiling. Several other bacterial taxa were also identified that could potentially be involved in different fertility disorders. Serological methods were not accurate enough since high cross-reactivity rates were observed. In the future, more accurate and efficient methods will be needed to determine the role of *M. canis* and its true role in the pathogenesis of specific fertility disorders in dogs.

## 1. Introduction

*Mycoplasma canis* (*M. canis*), like other bacteria of the Mollicutes class, is characterized by its small size, simplicity, and the absence of a cell wall. Due to their small genome size and the resulting limited ability to synthesize proteins, mycoplasmas live parasitically and are found in humans, mammals, reptiles, fish, arthropods, and even plants [1]. *M. canis* has already been isolated from dogs with clinical signs of urogenital disease and reproductive disorders [2,3,4,5,6,7]. Additional reports have demonstrated the presence of *M. canis* in atypical samples, such as nasal swabs and lung tissue samples of dogs suffering from canine infectious respiratory disease [8,9,10] and even in different human tissues following dog bites [11,12]. L’Abee-Lund et al. [2] have successfully isolated *M. canis* from the prostate, epididymis, and bladder wall. Maksimović et al. isolated *M. canis* and other mycoplasmas from the vagina of female dogs in different stages of the reproductive cycle [3]. Rosendal showed that experimental infection with *M. canis* led to various complications in the organs of the reproductive and urogenital tract of dogs [13].

Several reports state that mycoplasma species belonging to the genera *Mycoplasma*, *Ureaplasma*, and *Acholeplasma* are part of the normal mucosal microbiota [4,5,6,14,15]. *M. canis* has been isolated from mucosal surfaces of asymptomatic dogs as well as from dogs with non-specific clinical signs. The successful in vitro growth of *M. canis* isolated from clinical samples is hampered by the demanding growth requirements and the need for a nutrient-rich environment [16]. This also contributes to the lack of understanding of the role of specific virulence factors and the type of infection that *M. canis* causes in diseased animals [17]. We have previously identified hemagglutinins and neuraminidases as important virulence factors of canine mycoplasmas [18]. Differences in these factors influence the virulence in different strains. New knowledge of pathogen species-specific characteristics is important for setting up new investigation protocols; for example, molecular-based approaches. Reports on the presence of *M. canis* in samples from the canine urogenital and reproductive tract differ between serologic and molecular studies and a novel approach to diagnostics is needed.

In general, infertility in females is the inability to successfully conceive and give birth to offspring, even when mated repeatedly with a known fertile male at the time of ovulation. It is also defined as the inability to impregnate a fertile female at the time of ovulation. In some cases, presumed infertility is due to inadequate breeding management rather than the inability to conceive [19]. Knowledge of male infertility is inadequate. Poor semen quality can occur at any age, often due to unknown causes. Factors such as hormonal imbalances, heat, stress, toxins, or autoimmune diseases can contribute to infertility [20]. As many cases of infertility remain unexplained, we need to look at other possible causes of fertility issues, such as the composition of the microbiota of the female and male reproductive tract. So far, clinical signs of urogenital diseases and fertility problems cannot be directly linked to mycoplasma infection [21]. As reported by Ülgen et al., *M. canis* as well as other micro-organisms were isolated from urine samples, making it difficult to determine the causative agent and results have to be interpreted with caution [22]. Sometimes, infection can be subclinical and even when clinical signs are present, urogenital tract infections are primarily treated with broad-spectrum antibiotics, which have poor-to-no effect on mycoplasmas. The lack of a cell wall makes them resistant to several antibiotic species, such as penicillin, cephalosporin, vancomycin, and bacitracin [17]. The number of recent clinical studies is low, and the data of published studies are frequently contradictory [6,22,23,24]. Culture methods and serology are slowly being replaced by molecular methods, which are fast, have a lower detection limit, and offer high specificity [25,26,27,28]. The aim of this case study was to investigate whether fertility disorders in dogs can be linked to *M. canis* infection by comparing the results of several diagnostic techniques. Clinical examination findings were compared to the results of molecular and serological methods.

## 2. Materials and Methods

### 2.1. Dogs and Inclusion Criteria

A total of 30 dogs of variable breeds were included in this study. The dogs were divided into two groups. The first group included 14 dogs (10 females and 4 males) with various fertility disorders (fertility disorder group, FD) and the second group consisted of 16 clinically healthy dogs with a proven history of fertility and producing offspring (4 females and 12 males) (control group, CTRL). The dogs ranged from 1 to 13 years of age. All animals underwent routine clinical examination and hematological analysis. In addition, selected biochemical parameters and ultrasound examination were performed in all dogs.

The criteria for the inclusion of females in the FD group were as follows: inability to produce offspring (*n* = 8), abnormal cycle progression (*n* = 2), and spontaneous termination of pregnancy (*n* = 0). The criteria for the inclusion of males in the FD group were as follows: inability to copulate or ejaculate (*n* = 1), poor semen quality (*n* = 3), and disease of the prostate gland (*n* = 0). The CTRL group included dogs without chronic diseases that were treated during the survey and without fertility disorders as described above that have previously produced offspring.

### 2.2. Clinical Examination

All animals underwent a thorough clinical assessment (body condition score, evaluation of vital signs, genital discharge assessment, examination of the peripheral lymph nodes, detection of pain at abdomen palpitation), breeding soundness examination (for females: medical history, examination of the external genitalia, vaginoscopy, presence of discharge, assessment of mucous membranes; for males: medical history, assessment of libido, visual assessment of scrotum, palpation of the testes, examination of penis and prepuce), blood work, and ultrasound examination. Some male patients underwent semen evaluation. Vaginal or preputial swabs were collected from all patients.

#### 2.2.1. Hematological and Biochemical Analysis

Blood samples were collected by puncture of the peripheral vein (*v. cephalica*, *v. saphaena* or *v. jugularis*) into EDTA-containing tubes (Microtainer, Beckton and Dickinson, Franklin Lakes, NJ, USA) for the determination of complete blood count (CBC) and white blood cell differential count (WCDC). Routine hematological analysis was performed using an automated flow cytometry-based laser hematology analyzer (ADVIA 120, Siemens, Munich, Germany). For the determination of selected biochemical parameters (urea and creatinine concentration), blood serum was collected in 4 mL tubes with gel and added blood coagulation activator (Vacuette, Greiner Bio-One, Kremsmünster, Austria). Blood samples were kept at room temperature for 30 min and then centrifuged at 1300× *g* for 10 min at room temperature. Biochemical parameters were measured spectrophotometrically using an automated biochemical analyzer (RX Daytona, Randox, Crumlin, UK) with factory-made reagent kits (also Randox, Crumlin, UK). Sera protein and globulin levels were measured with the NanoDrop Lite instrument (Thermo Fischer Scientific, Waltham, MA, USA) using UV spectrophotometry designed to measure micro-volume amounts of proteins and globulins in the sera samples.

#### 2.2.2. Vaginal/Preputial Swab Collection and Cytology

Vaginal or preputial swabs were collected from all patients in triplicate using a sterile flocked swab with a tip coated with short Nylon fibers (Copan FLOQ Swabs, Brescia, Italy). Vaginal swabs were obtained from the cranial part of the vagina by first gently separating the vulvar lips with the fingers and then inserting the swab into the dorsal part of the vulva. The first and second replicate were stored at −70 °C for molecular analysis. One swab was used for DNA extraction and PCR-based detection of *M. canis* and the second swab was used for 16S microbiota profiling. The third replicate was immediately used for cytology. Dried smears were May–Grunwald Giemsa-stained and evaluated under a light microscope to determine the phase of the reproductive cycle and to check for the possible presence of inflammatory cells and bacteria.

#### 2.2.3. Semen Evaluation

Semen was collected from three out of four patients in the FD group (one dog had a complete lack of libido) and four patients in the CTRL group. Semen collection was performed by digital manipulation. Immediately after collection, the color, odor, consistency, and quantity of the collected semen were determined. The samples were also examined microscopically. Total and progressive motility of the spermatozoa in the sample were determined using computer-assisted CASA analysis (HTM-IVOS, version 12, Beverly, MA, USA). Prior to analysis, the samples were incubated at 37 °C for 10 min. The examination was conducted using a Makler chamber in which 5 μL of sample was dispensed. For each sample, two measurements were taken in three randomly selected fields, and their average values were used for calculations. Sperm concentration was determined with the CASA system and a Neubauer counting chamber. Cell membrane integrity was assessed using supravital eosin/nigrosine staining under a phase-contrast microscope at 1000× magnification. Fresh semen samples underwent morphological analysis and May–Grunwald Giemsa-stained smears were used to detect morphological changes. A total of 200 spermatozoa per subject were examined to determine the percentage of morphologically normal spermatozoa and the percentage of spermatozoa with individual morphological defects.

#### 2.2.4. Ultrasound Examination

Ultrasound examination of the urogenital system was performed using an 8C microconvex probe and an 11 L-D linear probe (both GE Healthcare, Chicago, IL, USA). In females, the ovaries, uterus, urinary bladder, and proximal urethra were examined. In males, the testes, prostate gland, urinary bladder, prostatic and penile urethra were examined.

### 2.3. Molecular Examination

#### 2.3.1. Nucleic Acid Extraction

Total nucleic acids were extracted from preputial and vaginal swabs. Prior to extraction, swabs were soaked in 2 mL of PBS (Dulbecco’s Phosphate Buffered Saline, Sigma-Aldrich, Burlington, MA, USA) and shaken for 1 min on a Bio Vortex V1 shaker (Biosan, Riga, Latvia). Nucleic acids were extracted from 140 μL of PBS solution using the QIAamp Viral RNA Mini Kit (Qiagen, Germantown, MD, USA) according to the manufacturer’s instructions. Nucleic acids were eluted in 60 μL elution buffer and stored at −70 °C until use.

#### 2.3.2. PCR-Based Detection of *M. canis*

Molecular detection of *M. canis* was performed using a conventional PCR based on the amplification of a 16S-23S rRNA gene intergenic spacer region (IGS), a 247 bp long species-specific nucleotide sequence. For this purpose, primer pair Myc1 and *M. canis*-R was used [29]. The 20 μL PCR reactions included 10 μL 2× DreamTaq Green PCR Master Mix (Thermo Scientific, Waltham, MA, USA), 1 μL (20 pmol) of each primer Myc1/*M. canis*-R, 2 μL of DNA, and deionized water. PCR amplification was performed using the GeneAmp PCR System 2700 (Applied Biosystems, Waltham, MA, USA). The cycle parameters included initial denaturation at 95 °C for 5 min, followed by 35 cycles of denaturation at 95 °C for 30 s, annealing at 55 °C for 30 s, elongation at 72 °C for 60 s, and a final elongation step at 72 °C for 5 min. Appropriate positive and negative controls were applied for each test.

All DNA samples were further tested to confirm the presence of *M. canis* and to obtain a partial neuraminidase gene sequence. Species-specific primer pair Ca-NEU-4F (5′ GGA ACA GCA ATT GAA AAT GAT CAC 3′) and Ca-NEU-4R (5′ TGT ATT CCG TTT CCT ACC CC 3′) targeting the 956 bp segment of the neuraminidase gene was used. This primer pair was constructed based on the sequence of the neuraminidase gene of the type strain *M. canis* PG14^T^ (MCANPG14_00438) [18]. Similarly, the 20 μL PCR reactions were performed using 10 μL 2× DreamTaq Green PCR Master Mix (Thermo Scientific, Europe), 0.5 μL (10 pmol) of each primer Ca-NEU-4F/Ca-NEU-4R, 2 μL of DNA, and deionized water. PCR amplification was performed using the GeneAmp PCR System 2700 (Applied Biosystems, Waltham, MA, USA) by applying the following parameters: initial denaturation at 95 °C for 5 min, followed by 35 cycles of denaturation at 95 °C for 30 s, annealing at 50 °C for 30 s, elongation at 72 °C for 75 s, and a final elongation step at 72 °C for 7 min.

#### 2.3.3. Purification of PCR Products and Sanger Sequencing

PCR products from both PCR reactions were separated on a 1.8% agarose gel (Sigma-Aldrich, Burlington, MA, USA) at 130 V for 30 min. The gel was stained with ethidium bromide (BioRad, Hercules, CA, USA) and the results were visualized using BioRad Gel Doc 2000 Imaging System on Quantity One 4.4.0 Analysis Software. PCR amplicons from the second PCR (primer pair Ca-NEU-4F/Ca-NEU-4R) of appropriate length were excised from the agarose gel and purified using the FastGene Gel/PCR Extraction Kit (Nippon Genetics Europe, Düren, Germany) according to the manufacturer’s instructions. The purified PCR amplicons were Sanger sequenced at Macrogen Europe (Amsterdam, The Netherlands). Sequences were analyzed using Finch TV 1.4.0 (Geospiza Inc., Seattle, WA, USA) and queried against the GenBank database [30] using BLASTn [31]. Obtained partial neuraminidase gene sequences were deposited into the GenBank database. A Clustal Omega alignment [32] of partial neuraminidase gene sequences was carried out, comparing the nucleotide sequences to other known mycoplasma neuraminidase genes, accessible in the GenBank database.

#### 2.3.4. 16S Microbiota Profiling

For 16S microbiota profiling, 16S rRNA gene amplicons were sequenced at Diversigen Inc. (New Brighton, MN, USA). For the analysis of the bacterial community composition in vaginal and preputial swabs, total DNA was extracted using the DNeasy PowerSoil Pro Kit automated for high throughput on QiaCube HT with bead beating in Qiagen Powerbed Pro plates (all Qiagen, Germantown, MD, USA). DNA was quantified using qPCR with primers targeting the hypervariable region V4 (515F/806R) of the 16S rRNA gene. Libraries were prepared with a protocol derived from Gohl et al. [33] using KAPA HiFi Polymerase (Roche Diagnostic, Basel, Switzerland) to amplify the hypervariable region V4 (515F/806R) of the 16S rRNA gene. 16S rRNA gene amplicons were sequenced on an Illumina MiSeq system using the paired-end (2 × 150 bp) mode and the MiSeq Reagent Kit v3 (both Illumina, San Diego, CA, USA).

DNA sequences were filtered for low quality (Q < 30) and length (<50 bases), and adapter sequences were trimmed using Cutadapt (Illumina, San Diego, CA, USA). FastQ files were converted to a single Fasta of stitched reads using shi7. Operational taxonomic units (OTUs) were picked using a closed reference approach at 97% identity against the Greengenes database using fully gapped alignment with Burst. Ties were broken by minimizing the overall number of unique OTUs. For taxonomy assignment, each input sequence was assigned the lowest common ancestor that was consistent across at least 80% of all reference sequences tied for best hit. Samples with fewer than 1000 sequences were discarded. Taxa that could not be unambiguously identified at a given taxonomic level were labeled as “Other”. Alpha diversity metrics were calculated from the filtered OTU table, which was rarefied to the minimum number of reads observed in all samples (*n* = 10,754) using QIIME 1.9.1 [34].

### 2.4. Serology

#### 2.4.1. Dot Immunobinding Assay

Dot immunobinding assay (DIBA) was used to detect the presence of antibodies against different mycoplasma proteins in dog sera. PVDF membrane with 0.45 μm pore size (Immobilon-P PVDF Membrane, Merck Millipore, Burlington, MA, USA) was activated in 100% methanol (Sigma-Aldrich, Burlington, MA, USA) and thoroughly washed in distilled water. Cell lysates of five different canine mycoplasma strains were used as antigen. Based on previous studies [18,35], the strains used were as follows: *M. canis* PG14^T^, *M. canis* Larissa, *M. cynos* 896, *M. cynos* 2296, and *M. molare* H542^T^. An amount of 2 μL of each antigen was dotted several times onto PVDF membrane and incubated in blocking buffer (0.5% Tween-PBS; Tween 20, Merck Millipore, Burlington, MA, USA) for 2 h at room temperature to block free antigen-binding sites. The membrane was cut up and individual membrane strips containing all five antigens were incubated in different dog sera, diluted to 1:100, for 1.5 h at room temperature. Strips were thoroughly washed three times for 10 min with 0.05% Tween-PBS and then further incubated in 1:2000 diluted HRP-conjugated rabbit anti-dog IgG antibodies (A6792, Sigma-Aldrich, Burlington, MA, USA) for 1 h at room temperature. Following another series of washing in 0.05% Tween-PBS for 10 min (2×) and PBS for 10 min (1×), the reactions were visualized using the TrueBlue chromogenic substrate (KPL, Gaithersburg, MD, USA).

#### 2.4.2. Western Blot Analysis

Proteins of whole-cell lysates of *M. canis* strains PG14^T^ (type strain) and Larissa (a highly virulent Slovenian isolate) were separated by polyacrylamide gel electrophoresis in the presence of sodium dodecyl sulfate (SDS-PAGE) using a modified Laemmli method [36]. Precast NuPAGE 4–12% Bis-Tris gels with 10 or 15 pockets (Thermo Fisher Scientific, Waltham, MA, USA) were used. Samples were diluted 1:1 with loading buffer (0.5 M Tris-HCl pH 6.8, 100% glycerol, 10% SDS, 0.25% bromophenol blue, and distilled water) and boiled at 95 °C for 10 min. Proteins were separated using an XCell SureLock Mini-Cell electrophoresis system (Thermo Fischer Scientific, Waltham, MA, USA) at 180 V for 60–70 min. PageRuler Plus Prestained Protein Ladder size standard (10–250 kDa, Thermo Fischer Scientific, Waltham, MA, USA) was used to track the electrophoresis flow and to compare the size of separated proteins on the PVDF membrane. After electrophoresis, the proteins were blotted onto a PVDF membrane using the Mini Trans-Blot Cell System (BioRad, Hercules, CA, USA) at 100 V for 90 min. the membrane was cut into strips, blocked for 2 h at room temperature, and incubated in dog sera (diluted 1:100) for 1.5 h at room temperature. Following washing (as described above), strips were incubated in 1:2000 diluted HRP-conjugated rabbit anti-dog IgG antibodies for 1 h at room temperature. Positive bands were visualized using the chromogenic substrate TrueBlue.

## 3. Results

### 3.1. Dogs and Inclusion Criteria

Demographic variables and dog breed had no marked impact on the presence of mycoplasma infection. Based on the inclusion criteria, dogs in the CTRL group showed no clinical signs pointing to mycoplasma infection or any kind of fertility disorder, even though some tested positive for the presence of mycoplasma by species-specific PCR.

### 3.2. Clinical Examination

All basic patient data, reproductive cycle assessment (females), clinical findings, ultrasound results, PCR, and 16S microbiota profiling results are presented in Table 1. No marked abnormalities were found during the clinical examination. One male and one female patient in the FD group with confirmed *M. canis* infection (by PCR) were treated with a recommended antibiotic (doxycycline, 5 mg/kg every 12 h for 7 days). After treatment, preputial and vaginal swab was tested again with species-specific PCR, which gave negative results.

#### 3.2.1. Hematological and Biochemical Analysis

All measured hematological and biochemical parameters and their values are shown in Table S1. For statistical evaluation of the data, one-way analysis of variance (ANOVA) was used. The level of statistical significance used was *p* < 0.05. No statistically significant discrepancies were found within the two study groups as well as separately for males and females. All measured parameters were within normal range, not showing any signs of bacterial infection or other abnormal health conditions.

#### 3.2.2. Cytology of Vaginal and Preputial Swabs

Cytology of vaginal swabs was used to differentiate the reproductive cycle phase in females (presented in Table 1). In the FD group, four females were in estrus, five in diestrus, and one in anestrus phase. In the CTRL group, two females were in estrus and two in diestrus phase. The cytological examination of swabs did not reveal the presence of abnormal cell structures, inflammatory cells, and no visible bacteria in tissue cells were detected. In the FD and CTRL group, the reproductive cycle phase in females showed no marked connection to mycoplasma infection. In males, no non-specific cells were found on cytological examination of the preputial swabs.

#### 3.2.3. Semen Evaluation

In the FD group, semen was successfully collected from three out of four patients. One male with a lack of libido had no ejaculate. In the three other patients, analysis revealed they had no sperm present in the seminal fluid (azoospermia); therefore, other semen parameters could not be assessed. In the CTRL group, CASA analysis and fresh semen smear analysis of four male patients showed no abnormalities. Results of the CASA analysis are presented in Table S2.

#### 3.2.4. Ultrasound Examination

Ultrasound examination was successfully performed in all 30 dogs included in the study (presented in Table 1). In females from the FD group, the collection of fluid in the uterus was observed in two patients and ovarian cysts were present in one patient. In one male patient from the FD group, cysts in the prostate were observed; this male had only one successful mating after several unsuccessful trials. In the CTRL group, one female had fluid in the uterus, four males had cysts in the prostate, one male suffered from benign prostatic hyperplasia (BPH), and two males suffered from BPH and simultaneously had a testes tumor present. However, all sexually mature animals in the CTRL group have previously mated successfully and produced healthy offspring and all changes were found incidentally in the scope of this study.

### 3.3. Molecular Examination

#### 3.3.1. PCR-Based Detection of *M. canis*

DNA from preputial and vaginal swabs of all thirty animals was successfully isolated and used to determine the presence of *M. canis* infection by PCR. Using the primer pair Myc1/*M. canis*-R, infection with *M. canis* was confirmed in 6/14 (42.9%) dogs in the FD group and 4/16 (25.0%) dogs in the CTRL group (Table 1). Patients 5 and 6 were tested again after a round of antibiotic treatment with doxycycline and their *M. canis*-specific PCR results were negative, pointing to a successful treatment strategy.

All DNA samples were used for a second PCR, in which a partial *M. canis* neuraminidase gene was amplified using the primer pair Ca-NEU-4F/Ca-NEU-4R. Neuraminidases of mycoplasma species are highly variable and offer a suitable positive control for a more precise determination. Even within the same species, some strains show high neuraminidase activity (e.g., *M. canis* Larissa), whereas some completely lack activity and the corresponding gene (*M. canis* LV) [18,37]. The amplification of the *M. canis* neuraminidase gene was successful in 6/14 (42.9%) dogs in the FD group and 4/16 (25.0%) dogs in the CTRL group (Table 1). In the FD group, all six patients were positive by both methods. Similarly, in the CTRL group, all four patients were positive by both methods.

#### 3.3.2. Partial Neuraminidase Gene Sequences

PCR amplicons obtained using the Ca-NEU-4F/Ca-NEU-4R primer pair were sent for Sanger sequencing. Partial *M. canis* neuraminidase gene sequences were successfully obtained in patients 3, 5, 6, 10, 13, 16, 19, 22, and 23. Partial gene sequences were deposited in GenBank with the following accession numbers: PP4300513, PP4300514, PP4300515, PP4300516, PP4300517, PP4300518, PP4300519, PP4300520, and PP4300521, respectively. The sequence of sample from patient 4 was not of sufficient quality. Nucleotide sequences revealed a 98–100% BLAST identity to other known sialidase sequences of several *M. canis* strains. Clustal Omega was used to produce a nucleotide alignment, which comprised the analyzed partial neuraminidase sequences and known sequences of *M. canis* strains Larissa, PG14^T^, UF31, UF33, UFG1, and UFG4 (accession numbers OP354259, JQ177148, AJFR01000002.1, AJFS01000002.1, AJFT01000002.1, and AJFU01000003.1, respectively) (Figure S1). The partial sequences contained several single nucleotide replacements. However, no nucleotide differences were present in important functional regions of the gene, for example, the RIP motif (TATCGTATCCCA, Figure S1, in yellow), which is present at the N-terminal of the corresponding protein and represents a part of the enzyme’s active site. Another prominent region in the neuraminidase gene is the Asp box motif (AGTTATGATGAAGGTAGAACATGA, Figure S1, in blue), a highly conserved domain that plays an important part in the catalytic function of the enzyme. No single nucleotide differences were present in this region.

#### 3.3.3. 16S Microbiota Profiling

**Figure 1:**
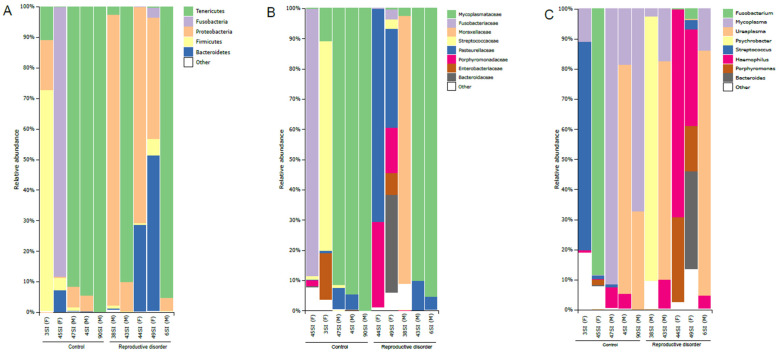
Microbiota composition of each sample. Samples are stratified according to the analyzed group: (**A**) top five bacterial phyla; (**B**) top eight bacterial families; (**C**) top eight bacterial genera. The sex of animals is indicated in brackets next to the sample name: F, female; M, male.

The samples for which the quality and quantity of DNA were insufficient for 16S microbiota profiling were excluded from the analysis. Samples with fewer than 1000 sequences were also excluded from the analysis. The final 16S microbiota profiling included 10/30 (33.3%) animals, namely 5/14 (35.7%) dogs from the FD group and 5/16 (31.3%) dogs from the CTRL group. Community composition for each sample at different taxonomic levels is presented in Figure 1 and individual species composition for each patient is presented in Table S3. Different species from the genus *Mycoplasma* were detected in 3/5 (60.0%) samples from the FD group and 4/5 (80.0%) samples from the CTRL group. Other most-represented genera included *Ureaplasma*, *Psychrobacter*, *Streptococcus*, *Fusobacterium*, *Haemophilus*, *Porphyromonas*, and *Bacteroides* (Figure 1). *M. canis* was detected in patients 6, 16, 17, 23, and 43 (2.48%, 10.77%, 0.01%, 0.23%, and 0.24%, respectively). Patient 6 from the FD group and patients 16 and 23 from the CTRL group were *M. canis*-positive by both the species-specific PCR and 16S microbiota profiling.

The relative abundance of the family *Mycoplasmataceae* did not differ significantly between the study groups (*p* = 0.2343, Mann–Whitney *U* test). The relative abundance of the genus *Mycoplasma* also did not differ significantly between the study groups (*p* = 0.1425, Mann–Whitney *U* test). However, when dogs from both study groups were stratified by sex, the relative abundance of the genus *Mycoplasma* was significantly higher in males than in females (*p* = 0.0247, Mann–Whitney *U* test). In addition, the two samples with the highest relative abundance of *Mycoplasma* originated from males in the CTRL group (Figure 1).

**Figure 2:**
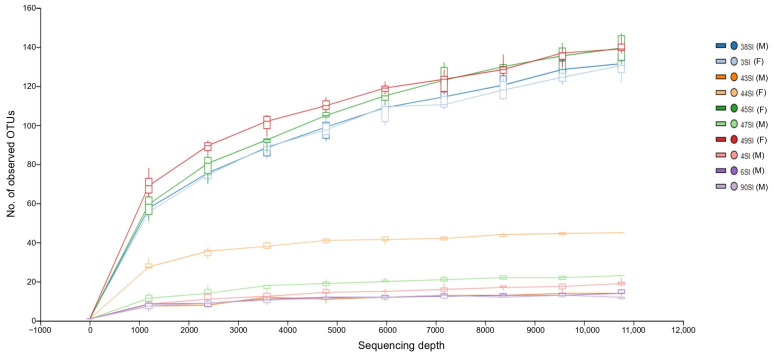
Rarefaction curves showing the number of OTUs per sequencing depth. The sex of the animal is indicated in brackets next to the sample name: F, female; M, male.

Alpha diversity indices (Chao1, Shannon, and Simpson indices, as well as the number of observed OTUs) for the ten analyzed samples are reported in Table S4. The number of observed OTUs ranged from 12 to 138. The rarefaction curves reached the near plateau phase, suggesting that the sequencing depth employed in this study reflects well the total bacterial diversity present in the studied samples, as shown in Figure 2. The number of observed OTUs did not differ significantly between the study groups (*p* = 1.000), but was significantly higher in females than in males (*p* = 0.0422), as indicated by the Mann–Whitney *U* test.

### 3.4. Serology

Blood serum was successfully collected from all 30 animals included in this study. Additionally, for patients 5 and 6, who received antibiotic treatment, serum after treatment was also collected and tested with serological methods. DIBA was used as a preliminary method for Western blot, to investigate the presence of mycoplasma-specific antibodies in all 32 sera. Five canine mycoplasma strains showing variable virulence in a previous study [18] were used as the antigen (*M. canis* PG14^T^, *M. canis* Larissa, *M. cynos* 896, *M. cynos* 2296, and *M. molare* H542^T^). In the FD group, all sera reacted with *M. canis* Larrisa proteins and 7/14 sera (50.0%) reacted with *M. canis* PG14^T^ proteins. A large proportion of sera also showed a reaction to the *M. cynos* and *M. molare antigens*, respectively. In total, 9/14 (64.3%) sera reacted to *M. cynos* 896, 5/14 (35.7%) sera to *M. cynos* 2297, and 5/14 (35.7%) to *M. molare* H542^T^. When compared to PCR results, we can see that even animals, that were PCR negative, showed one or more positive reaction to mycoplasma antigens. Similarly, in the CTRL group, a large number of sera reacted to more than one mycoplasma antigen. Only one patient sera from the CTRL group was completely negative in DIBA (patient 15). In total, 15/16 (93.8%) sera reacted with the *M. canis* Larrisa antigen, 12/16 (75.0%) sera reacted with *M. canis* PG14^T^, 13/16 (81.3%) sera reacted to *M. cynos* 896, 8/16 (50.0%) sera to *M. cynos* 2297, and 7/16 (43.8%) to *M. molare* H542^T^. DIBA may be a good indicative method when researching the prevalence of infection during the whole lifespan of the animal. However, it does not differentiate between acute and chronic infections, or active or past infections, and we must always consider the possibility of antibody cross-reactivity, especially in closely related strains or evolutionary highly conserved proteins. The two sera collected after treatment also reacted to several mycoplasma antigens in DIBA, whereas the post-treatment PCR results were negative.

**Figure 3:**
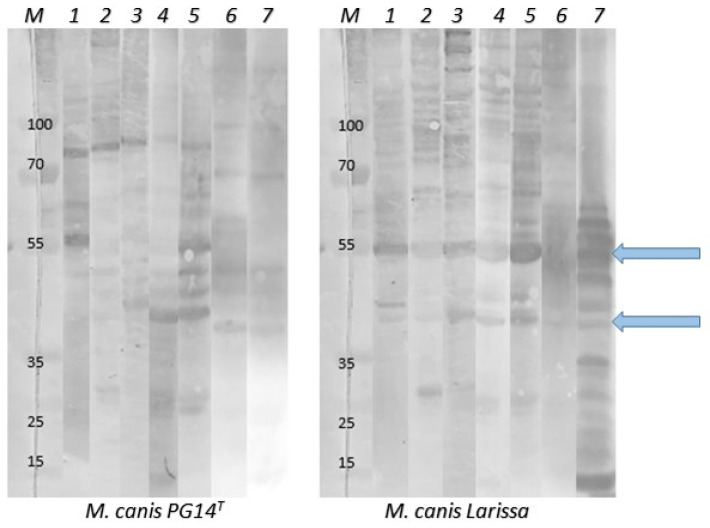
The presence of *M. canis*-specific antibodies in selected sera of the analyzed animals. Whole cell lysates of *M. canis* PG14^T^ (left panel) and *M. canis* Larissa (right panel) were incubated in sera of animals from the FD and CTRL group. Reactions of serum antibodies with mycoplasma proteins, ranging from 15 to 130 kDa, are clearly visible in all samples, regardless of the results of molecular examination. In particular, in the right panel (*M. canis* Larissa), antibodies reacted to two proteins, approximately 55 and 40 kDa in size (blue arrows). M: molecular size marker, PageRuler Plus Prestained Protein Ladder), 1: serum of patient 1/FD, 2: serum of patient 6/FD, 3: serum of patient 11/FD, 4: serum of patient 13/FD, 5: serum of patient 16/CTRL, 6: serum of patient 42/CTRL, 7: serum of patient 5/FD after antibiotic treatment.

In Western blot, *M. canis* PG14^T^ and Larissa proteins, derived from whole cell lysates, were blotted onto a PVDF membrane and incubated in dog sera. All fourteen sera from the FD group, nine sera from the CTRL group, and two sera after treatment were used. The antibodies present in all the tested sera bound to several different mycoplasma proteins, ranging from 15 to 130 kDa (Figure 3). The range of reacting proteins varies between *M. canis* PG14^T^ and *M. canis* Larissa cell lysates, as shown in Figure 3. This corresponds to several differences between the two strains as observed in our previous study [18]. In all sera from the FD group, there were two prominent reactions to proteins of approximately 55 and 40 kDa. Even the animals without clinical signs of infection or with negative PCR results produced many antigen–antibody immune reactions.

## 4. Discussion

*Mycoplasma canis* is a well-known opportunistic canine pathogen commonly associated with lower urinary tract infections and fertility problems [2,3,16,22]. Although the first reports of *M. canis* date back to the 1970s [5,38], many questions about the possible virulence factors that play a crucial role in the establishment of infections, the host immune responses they trigger, and their precise role in long-term disease pathogenesis remain unanswered. The prevalence of mycoplasma infections in dogs is underestimated and difficult to assess. Infections are often subclinical, i.e., do not cause visible health problems with pronounced clinical signs, and standard antibiotic treatment is ineffective, due to the lack of a cell wall [1]. In this study, we aimed to evaluate the value of clinical signs in the diagnosis of *M. canis* infection, the usability of routinely collected diagnostic parameters for early detection of infection, and the accuracy of an individual detection method. We wanted to gain a better understanding of how *M. canis* detection methods could be improved in relation to canine reproductive issues to provide more reliable diagnostic and treatment options in the future.

The treatment of canine fertility disorders is a complex process in which the veterinarian must take into account various causes and have access to a complete medical history, supplemented by a thorough clinical examination. Fertility is a complex biological process, and issues are often a result of multiple factors [6,39,40,41]. In addition, anamnestic data obtained from owners can be biased. The detection of mycoplasma is rarely requested in clinical samples from dogs and few laboratories worldwide routinely perform mycoplasma cultures. Reports on the detection methods used and the results obtained vary widely from one study to another. Certain species, like *M. haemocanis*, have so far only been identified using molecular techniques [42]. Chalker et al. [25] found that 15% of lung tissue extracts were PCR-positive, but the presence of mycoplasma in these samples was not confirmed by culture methods. At the same time, they found that 20% of culture-positive samples were negative by PCR, which is currently considered the most effective method, especially since it is more sensitive than culture. However, it should be noted that PCR does not prove whether bacteria are alive or not [9,43], except when using viability PCR [44]. It is still unclear which factors have a direct influence on susceptibility to mycoplasma infections. It has not been proven that demographic variables influence susceptibility to infection. However, it is plausible that these variables have an indirect effect that is difficult to determine or measure. For example, age may influence the animal’s immune status, which could allow mycoplasmas to evade the immune system. In this study, we could not connect age, sex, breed, and body weight of the dog with the presence of *M. canis* infection. Furthermore, *M. canis* infection was not related to the stage of the reproductive cycle, as previously reported [3,14]. Because blood and urine samples are relatively non-invasive samples that are routinely collected during veterinary examinations, we attempted to identify possible deviations of the blood or urine parameters from the reference values. This would allow rapid confirmation of infection. However, there were no conspicuous abnormalities in hematologic or biochemical parameters that could indicate the possible presence of *Mycoplasma* sp. Ultrasound anomalies were equally present in both groups and could not be associated with mycoplasma infection. There were evident differences in male semen evaluation between the dogs in the FD and CTRL groups, but the number of patients examined was small. Patients in the FD group had no sperm present in the seminal fluid, while males in the CTRL group had normal sperm on examination. Dogs can be asymptomatic carriers of mycoplasma, and the present clinical examination data seem to support the theory of a subclinical host colonization-type infection.

Another problem is the limited number of reports focusing on the incidence of cultivation of *M. canis* (and other canine mycoplasmas) in clinically healthy individuals. The results of available studies are inconsistent and often contradictory. It has already been shown that *M. canis* is present in clinically healthy dogs as well as in dogs with fertility issues and urogenital disease [3,5,6,22,24,45]. To date, no consensus has been reached as to whether *M. canis* is the cause of urogenital problems or simply appears as part of the normal microbiota. Other mycoplasmas that have been described and isolated from the urogenital tract of healthy and diseased animals are *M. cynos*, *M. spumans*, *M. maculosum*, *M. edwardii,* and *M. molare* [13,16]. In a study by Maksimovič et al. [3], *M. canis* was the most frequently isolated mycoplasma (vaginal swabs) in domestic and stray, intact and spayed females. In the same study, the stages of the reproductive cycle did not appear to affect the number or type of positive isolates. *M. canis* and other canine mycoplasmas were not detected in the uterus. If *M. canis* is indeed part of the normal microbiota in the urethra and uterus, the results of culture-based detection in dogs should be interpreted with caution. Ülgen et al. [22], for example, showed that *M. canis* can be cultured from swabs from dogs with urinary tract infections, but also from urinary tract mucosal samples from healthy dogs. L’Abee-Lund et al. [2] showed that *M. canis* is probably responsible for inflammatory changes in dogs with signs of urogenital disease, as *M. canis* was usually isolated in pure culture and the signs of infection only subsided after treatment with tetracycline, while all previous treatments were unsuccessful. According to the current knowledge, urinary tract or genital infections caused by mycoplasma cannot be differentiated from infections caused by other micro-organisms. Potential new players should also be considered. In a recent report, *M. maculosum* and *M. spumans*, but not *M. canis*, were associated with fertility problems in kennel dogs. One male dog had a low sperm count and low sperm motility, and one female dog was PCR-positive for *M. maculosum* and *M. spumans*. After treatment with doxycycline, the sperm count normalized, and successful pregnancies were achieved after additional treatment with azithromycin [23].

There is an existing need to introduce newer methods to clearly confirm the presence of mycoplasma in canine reproductive tract samples. Culturing is no longer the method of choice to determine the presence of mycoplasma due to the need for selective growth media and the low reproducibility of results. Researchers are trying to introduce new, culture-independent methods for mycoplasmas. Mycoplasmas can be firmly attached to mucosal surfaces and can easily be outcompeted by other, faster-growing micro-organisms. A suitable replacement lies in the precise development of various molecular-based methods.

PCR is a fast, reliable, and sensitive method, that is used in laboratories all over the world. Combined with DNA extraction from vaginal and preputial swabs, which is a non-invasive sampling option, it is the perfect method of choice for the clinical setting. In this study, 10/30 (33.3%) of the included animals were *M. canis-*positive by PCR. However, positive samples were present in the FD and CTRL groups, supporting the hypothesis that *M. canis* is part of the normal mucosal microbiota. It would be very helpful to determine existing molecular differences between *M. canis* isolates from healthy and diseased animals. This would potentially give us much-needed insights into the pathogenicity patterns of different virulent strains. We have already identified a highly virulent *M. canis* strain Larissa (Slovenian isolate) which showed high titers in hemagglutination and hemagglutination inhibition tests and much more prominent neuraminidase enzymatic activity compared to some less virulent strains (PG14^T^, UFG4, UF31, LV, and B16/08) [18]. In the future, it would be advantageous to identify these differences at the molecular (gene) level and use them for more precise diagnostics.

To further confirm the involvement of *M. canis* in fertility issues, DNA from vaginal and preputial swabs was subjected to 16S microbiota profiling. This method provides information on the overall composition of the bacterial community present in a sample and indicates potential (new) pathogens. This method is complex and involves many steps that can influence the results such as sample collection and storage, DNA extraction and amplification, sequencing, and analyses of obtained results, especially in low-biomass samples [46]. However, species-specific identification based on sequencing of the V4 region of the 16S rRNA gene is difficult and does not provide definitive confirmation that a particular pathogen species is also the cause of a disorder or disease. Furthermore, this method is too time-consuming and costly for everyday clinical practice to be considered the method of choice. In this study, only a small proportion of the samples subjected to 16S microbiota profiling provided sufficient genetic material to be included in the final analysis (10/30). If more than one swab sample is taken, it is very difficult to ensure the uniformity of the samples, which must be considered. In addition, different swab types can affect the DNA yield. A better success rate was previously observed by Lyman et at. [47], who were able to amplify 16S rRNA fragments from each sample collected from 50 female dogs using culturette swabs. However, in three samples that were positive for *M. canis* by PCR, the presence of *Mycoplasma* sp. was also confirmed by 16S microbiota profiling (Table 1 and Table S3). We also confirmed the presence of several other bacterial species in dogs from the FD and CTRL groups, including *Pseudomonas* sp., *Psychrobacter* sp., *Fusobacterium* sp., *Streptococcus* sp., and several other mycoplasma species (*M. cynos*, *M. spumans*, *M. opalescens*, and *M. fermentans*). Similarly, Burton et al. [24] identified several other bacterial strains in the genital microbiome of healthy dogs. Further investigation is needed to determine whether any of these strains are associated with fertility challenges.

Another source of information is the use of serological methods to detect specific antibodies. By collecting blood sera from canine patients, we were able to perform serological tests. We hoped to be able to relate data on the presence of specific antibodies against mycoplasma in canine sera to the molecular data we obtained. However, most of the dog sera used for DIBA and Western blot analyses contained specific and non-specific antibodies and reacted in many reactions against *M. canis* and the cell lysates of mycoplasma strains used. There has long been a problem with cross-reactivity between different mycoplasma strains in serology. Cross-reactivity has been previously demonstrated [26,38] and serologic results should be interpreted with caution, especially when no titration of sera was performed and no paired samples collected a few weeks apart were collected. Furthermore, the presence of antibodies in serum indicates that the animal has been in contact with a particular bacterial strain at a certain time, but this does not reflect the current infection status. Nevertheless, serologic testing can help us gain insight into possible virulence factors. We have detected antibody responses to various *M. canis* proteins, with responses to 55 and 40 kDa proteins being most prominent in the *M. canis* Larissa cell lysate. Similarly, we have previously identified novel virulence factors in *M. cynos* [35].

This study has several limitations that should be considered when planning new, more in-depth studies. Two of the most important limitations are the small number of animals included and the imbalance between male and female representatives in both study groups. Future studies need to focus on a larger number of subjects of both sexes to gain new insights into clinical symptoms and microbiota composition to better assess the relationship between mycoplasma load and clinical signs in males and females. A large number of animals could also help to gain new insights into inter-individual variability in health and disease. Identifying the causes of strain variability and differences in virulence may help us find new markers of pathogenicity. Further characterization of strains in a cohort with the development of more reliable techniques is necessary, and an in-depth knowledge of the diversity of their phylogenetic origin and taxonomy is required for a correct interpretation of the results obtained. Studying the molecular structure of individual strains and their exact mechanism of pathogenicity will give us more information on how to successfully combat the infection. In the future, we should focus on the individual virulence factors and how they can be used for targeted detection. Further studies are needed to clarify how these organisms evade the host’s immune defenses. As molecular methods advance, we are gaining new insights into inherent microbial communities and the complex relationships behind some often-unexplained health problems in veterinary medicine.

To successfully combat mycoplasma-related fertility problems in dogs, it is essential to raise awareness of this problem among owners and breeders and thereby increase the number of dogs tested. We recommend that *M. canis*-positive dogs with fertility problems are treated according to current guidelines. As mycoplasma can be part of the commensal microbiota, preventive treatment of animals without fertility problems remains controversial, especially in view of increasing antibiotic resistance and possible drug side effects. Future studies are needed to establish clinical standards and determine the cause of action for *M. canis*-positive dogs without fertility problems.

## Figures and Tables

**Table 1:** List of patients included in this study, denoting key demographic values, reproductive cycle assessment, main clinical findings, PCR and 16S microbiota profiling results.

Study Group	Patient Number	Breed	Sex	Age (years)	Body Weight (kg)	Phase of Estrous Cycle	Clinical Findings	Ultrasound Exam	PCR Results		16S Microbiota Profiling
									* Myc1/ * *M. canis-R*	*Ca-NEU-4F/* *Ca-NEU-4R*	
**FD group**	1	Parson Russel terrier	M	6	4.7	NA	Poorly expressed libido, never had offspring	Unremarkable	−	−	Done
	2	Bernese mountain dog	M	4	36.2	NA	Successfully mated only once	Cysts in the prostate	−	−	NS
	3	German shepherd	F	6	29.3	Estrus	Regularly in heat, mating never successful	Unremarkable	+	+/s	NS
	4	German shepherd	F	5	26.1	Diestrus	Last mating unsuccessful, only once with offspring	Unremarkable	+	+	NS
	5	Golden retriever	F	4	34.2	Estrus	Regularly in heat, mating never successful	Unremarkable	+	+/s	NS
	6	Golden retriever	M	5	36.0	NA	No libido	Unremarkable	+	+/s	Done
	7	Airedale terrier	F	8	28.3	Diestrus	Last two matings not successful	Fluid in the uterus	−	−	NS
	8	Airedale terrier	M	5	30.1	NA	Poorly expressed libido, only once with offspring	Unremarkable	−	−	Done
	9	German shepherd	F	4	28.3	Estrus	Regularly in heat, mating never successful	Unremarkable	−	−	Done
	10	American Staffordshire terrier	F	3	20.1	Diestrus	Regularly in heat, mating never successful	Unremarkable	+	+/s	NS
	11	Dogo Argentino	F	6	30.2	Estrus	Last two matings not successful	Ovarian cysts	−	−	Done
	12	Bernese mountain dog	F	2	38.3	Diestrus	Last two matings not successful	Unremarkable	−	−	NS
	13	Bernese mountain dog	F	3	40.2	Diestrus	Last two matings not successful	Unremarkable	+	+/s	NS
	14	Weimaraner	F	3	24.9	Anestrus	Regularly in heat, mating never successful	Fluid in the uterus	−	−	NS
**CTRL group**	15	English springer spaniel	F	9	17.4	Estrus	Unremarkable	Unremarkable	−	−	NS
	16	Cross breed	F	1	21.0	Diestrus	Unremarkable	Unremarkable	+	+/s	Done
	17	Japanese chin	M	3	3.1	NA	Unremarkable	Unremarkable	−	−	Done
	19	Scottish terrier	M	7	8.6	NA	Unremarkable	Cysts in the prostate	+	+/s	NS
	20	Scottish terrier	M	1	10.4	NA	Unremarkable	Unremarkable	−	−	NS
	21	Rottweiler	M	10	42.3	NA	Unremarkable	BPH and testes tumor	−	−	NS
	22	Rottweiler	M	2	45.0	NA	Unremarkable	Cysts in the prostate	+	+/s	NS
	23	Dobermann	M	4	39.5	NA	Unremarkable	BPH and testes tumor	+	+/s	Done
	29	Borzoi	M	3	39.6	NA	Unremarkable	Cysts in the prostate	−	−	NS
	32	Cross breed	M	10	28.7	NA	Unremarkable	BPH	−	−	NS
	33	Borzoi	M	1	42.4	NA	Unremarkable	Unremarkable	−	−	NS
	40	Golden retriever	M	6	33.0	NA	Unremarkable	Unremarkable	−	−	NS
	41	Golden retriever	F	4	28.7	Estrus	Unremarkable	Unremarkable	−	−	NS
	42	Cross breed	F	13	14.7	Diestrus	Unremarkable	Fluid in the uterus	−	−	Done
	43	Maltese dog	M	7	4.8	NA	Unremarkable	Cysts in the prostate	−	−	Done
	44	Weimaraner	M	8	33.0	NA	Unremarkable	Unremarkable	−	−	NS

M, male; F, female; NA, not applicable; BPH, benign prostatic hyperplasia; s, partial neuraminidase gene sequence; NS, not successful; +, positive result; −, negative result.

## Data Availability

Partial *M. canis* neuraminidase sequences are available in GenBank under accession number PP430513, PP430514, PP430515, PP430516, PP430517, PP430518, PP430519, PP430520, and PP430521.

## Supplementary Materials

The following supporting information can be downloaded at: https://www.mdpi.com/article/10.3390/pathogens13050391/s1, Figure S1: Clustal Omega alignment of the nine partial *M. canis* neuraminidase gene sequences; Table S1: Representation of all numerical values of monitored hematological and biochemical parameters for patients in FD and CTRL group; Table S2: Sperm parameters of four males from CTRL group. Table S3: Results of 16S microbiota profiling; Table S4: Alpha diversity metrics of the 10 samples that were included in the final 16S microbiota profiling.
