# Supplementary material for: Association of Mycoplasma canis with Fertility Disorders in Dogs: A Case Study Supported by Clinical Examination, PCR, 16S Microbiota Profiling, and Serology
Source: Pathogens. 2024 May 8;13(5):391. doi: 10.3390/pathogens13050391 (PMC11123722; doi:10.3390/pathogens13050391)
Supplement: Supplementary file 1 [file pathogens-13-00391-s001.zip › Figure S1.pdf]

Figure S1. Clustal Omega alignment of the nine partial *M. canis* neuraminidase gene sequences (denoted with number corresponding to patient number). Selected publicly available and annotated neuraminidase gene sequences of other *M. canis* strains were included for comparison.

```
Larissa      ATGAAAAAAATTAATAAAAAATTAGCTGTATATCTGCTCTTATCAGGAGCACTTATAACA      60
16           ------------------------------------------------------------      0
10           ------------------------------------------------------------      0
UFG1         ATGAAAAAAATTAATAAAAAATTAGCTGTATATCTGCTCTTATCAGGAGCACTTATAACA      60
3            ------------------------------------------------------------      0
UF31         ATGAAAAAAATTAATAAAAAATTAGCTGTATATCTGCTCTTATCAGGAGCACTTATAACA      60
UF33         ATGAAAAAAATTAATAAAAAATTAGCTGTATATCTGCTCTTATCAGGAGCACTTATAACA      60
PG14T        ATGAAAAAAATTAATAAAAAATTAGCTGTATATCTGCTCTTATCAGGAGCACTTATAACA      60
23           ------------------------------------------------------------      0
UFG4         ATGAAAAAAATTAATAAAAAATTAGCTGTATATCCGCTCTTATCAGGAGCACTTATAACA      60
22           ------------------------------------------------------------      0
13           ------------------------------------------------------------      0
19           ------------------------------------------------------------      0
5            ------------------------------------------------------------      0
6            ------------------------------------------------------------      0


Larissa      TCGACTTCTTTAGCAATATATTTCTATGCAAATAGTCAAAAGATTGAGAAAAAGAAGAAG      120
16           ------------------------------------------------------------      0
10           ------------------------------------------------------------      0
UFG1         TCGACTTCTTTAGCAATATATTTCTATGCAAATAGTCAAAAGATTGAGAAAAAGAAGAAG      120
3            ------------------------------------------------------------      0
UF31         ACGACTTCTTTAGCAATATATTTCTATGCAAATAGTCAAAAGATTGAGAAAAAGAAGAAG      120
UF33         TCGACTTCTTTAGCAATATATTTCTATGCAAATAGTCAAAAGATTGAGAAAAAGAAGAAG      120
PG14T        TCGACTTCTTTAGCAATATATTTCTATGCAAATAGTCAAAAGATTGAGAAAAAGAAGAAG      120
23           ------------------------------------------------------------      0
UFG4         TCGACTTCTTTAGCAATATATTTCTATGCAAATAGTCAAAAGATTGAGAAAAAGAAGAAG      120
22           ------------------------------------------------------------      0
13           ------------------------------------------------------------      0
19           ------------------------------------------------------------      0
5            ------------------------------------------------------------      0
6            ------------------------------------------------------------      0


Larissa      AGCAATTCATTTTCTGATAGTCAAATTAATGGTTTTGAATTTAACATCCCTGAAAAACAT      180
16           ------------------------------------------------------------      0
10           ------------------------------------------------------------      0
UFG1         AGCAATTCATTTTCTGATAGTCAAATTAATGGTTTTGAATTTAACATCCCTGAAAAACAT      180
3            ------------------------------------------------------------      0
UF31         AGCAATTCATTTTCTGATAGTCAAATTAATGGTTTTGAATTTAACATCCCTGAAAAACAT      180
UF33         AGCAATTCATTTTCTGATAGTCAAATTAATGGTTTTGAATTTAACATCCCTGAAAAACAT      180
PG14T        AGCAATTCATTTTCTGATAGTCAAATTAATGGTTTTGAATTTAACATCCCTGAAAAAGAT      180
23           ------------------------------------------------------------      0
UFG4         AGCAATTCATTTTCTGATAGTCAAATTAATGGTTTTGAATTTAACATCCCTGAAAAACAT      180
22           ------------------------------------------------------------      0
13           ------------------------------------------------------------      0
19           ------------------------------------------------------------      0
5            ------------------------------------------------------------      0
6            ------------------------------------------------------------      0
```

```
Larissa     AATACTTTTGTTCCTAACAATAATAACCATAACGACTTTATTGAGCAACCTGAAAAAAGT     240
16          ------------------------------------------------------------     0
10          ------------------------------------------------------------     0
UFG1        AATACTTTTGTTCCTAACAATAATAACCATAACGACTTTATTGAGCAACCTGAAAAAAGT     240
3           ------------------------------------------------------------     0
UF31        AATACTTTTGTTCCTAACAATAATAACCATAACGACTTTATTGAGCAACCTGAAAAAAGT     240
UF33        AATATTTTTGTTCCTAACAATAATAACCATAACGACTTTATTGAGCAACCTGAAAAAAGT     240
PG14T       AATACTTTTGTTCCTAACAATAATAACCATAACGACTTTATTGAGCAACCTGAAAAAAGT     240
23          ------------------------------------------------------------     0
UFG4        AATACTTTTGTTCCTAACAATAATAACCATAACGACTTTATTGAGCAACCTGAAAAAAGT     240
22          ------------------------------------------------------------     0
13          ------------------------------------------------------------     0
19          ------------------------------------------------------------     0
5           ------------------------------------------------------------     0
6           ------------------------------------------------------------     0

Larissa     GATGTCAAATTTTATAACTTTCAATTCAAAAAAAGTGATGTCGATGATTCTTCGTTTATA     300
16          ------------------------------------------------------------     0
10          ------------------------------------------------------------     0
UFG1        GATGTCAAATTTTATAACTTTCAATTCAAAAAAAGTGATGTCGATGATTCTTCGTTTATA     300
3           ------------------------------------------------------------     0
UF31        GATGTCAAATTTTATAACTTTCAATTCAAAAAAAGTGATGTCGATGATTCTTCGTTTATA     300
UF33        GATGTCAAATTTTATAACTTTCAATTCAAAAAAAGTGATGTCGATGATTCTTCGTTTATA     300
PG14T       GATGTCAAATTTTATAACTTTCAATTCAAAAAAAGTGATGTCGATGATTCTTCGTTTATA     300
23          ------------------------------------------------------------     0
UFG4        GATGTCAAATTTTATAACTTTCAATTCAAAAAAAGTGATGTCGATGATTCTTCGTTTATA     300
22          ------------------------------------------------------------     0
13          ------------------------------------------------------------     0
19          ------------------------------------------------------------     0
5           ------------------------------------------------------------     0
6           ------------------------------------------------------------     0

Larissa     AGTTTGAATTTTGAAGGTACTAAACTTTTATCAGAATCAAGATTACAAATTGAACTTGAG     360
16          ------------------------------------------------------------     0
10          ------------------------------------------------------------     0
UFG1        AGTTTAAATTTTGAAGGTACTAAACTTTTATCAGAATCAAGGTTACAAATCGAACTCGAG     360
3           ------------------------------------------------------------     0
UF31        AGTTTAAATTTTGAAGGTACTAAACTTTTATCAGAATCAAGATTACAAATCGAACTCGAG     360
UF33        AGTTTAAATTTTGAAGGTACTAAACTTTTATCAGAATCAAGGTTACAAATCGAACTCGAG     360
PG14T       AGTTTAAATTTTGAAGGTACTAAACTTTTATCAGAATCAAGATTACAAATTGAACTTGAG     360
23          ------------------------------------------------------------     0
UFG4        AGTTTAAATTTTGAAGGTACTAAACTTTTATCAGAATCAAGATTACAAATTGAACTTGAG     360
22          ------------------------------------------------------------     0
13          ------------------------------------------------------------     0
19          ------------------------------------------------------------     0
5           ------------------------------------------------------------     0
6           ------------------------------------------------------------     0
```

```
Larissa        GACAACAATAAAAATTTAGTCGTTTTAGATAAGTTTAAAATCAATAACGAGAACAAGGAA      420
16             ------------------------------------------------------------      0
10             ------------------------------------------------------------      0
UFG1           GACAACAATAAAAATTTAGTCGTTTTAGATAAGTTCGAGATCAATAACGAAAGCAAGGAA      420
3              ------------------------------------------------------------      0
UF31           GACAACAATAAAAATTTAGTCGTTTTAGATAAGTTTAAAATCAATAACGAAAACAAGGAA      420
UF33           GACAACAATAAAAATTTAGTCGTTTTAGATAAGTTCGAGATCAATAACGAAAGCAAGGAA      420
PG14T          GACAACAATAAAAATTTAGTCGTTTTAGATAAGTTTAAAATCAATAACGAGAACAAGGAA      420
23             ------------------------------------------------------------      0
UFG4           GACAACAATAAAAATTTAGTCGTTTTAGATAAGTTTAAAATCAATAACGAGAACAAGGAA      420
22             ------------------------------------------------------------      0
13             ------------------------------------------------------------      0
19             ------------------------------------------------------------      0
5              ------------------------------------------------------------      0
6              ------------------------------------------------------------      0


Larissa        GTTGTTTTTGATACTAGTGATCTAACTAATAACAGAAAGTTCAATGTCAAGACAATTACT      480
16             ------------------------------------------------------------      0
10             ------------------------------------------------------------      0
UFG1           GTTGTTTTTGATGCTAGTGATCTAACTAATAACAGAAAGTTCAATGTCAAGACAATTACT      480
3              ------------------------------------------------------------      0
UF31           GTTGTTTTTGATACTAGTGATCTAACTAATAACAGAAAGTTCAATGTCAAGACAATTACT      480
UF33           GTTGTTTTTGATGCTAGTGATCTAACTAATAACAGAAAGTTCAATGTCAAGACAATTACT      480
PG14T          GTTATTTTTGATACTAGTGATCTAACTAATAATAGAAAGTTCAATGTCAAGACAATTACT      480
23             ------------------------------------------------------------      0
UFG4           GTTGTTTTTGATACTAGTGATCTAACTAATAACAGAAAGTTCAATGTCAAGACAATTACT      480
22             ------------------------------------------------------------      0
13             ------------------------------------------------------------      0
19             ------------------------------------------------------------      0
5              ------------------------------------------------------------      0
6              ------------------------------------------------------------      0


Larissa        TTAAATGATCAATTAATTAAAAATGATAACGACATTGATGATGATGCTGAGAAAATTTCA      540
16             ------------------------------------------------------------      0
10             ------------------------------------------------------------      0
UFG1           TTAAATGATCAATTAATTAAAAATGATAACGACATTGATGATGATACTGAGAAAATTTCA      540
3              ------------------------------------------------------------      0
UF31           TTAAATGATCAATTAATTAAAAATGATAACGACATTGATGATGATACTGAGAAAATTTCA      540
UF33           TTAAATGATCAATTAATTAAAAATGATAACGACATTGATGATGATACTGAGAAAATTTCA      540
PG14T          TTAAATGATCAATTAATTAAAAATGATAACGACATTGATGATGATGCTGAGAAAATTTCA      540
23             ------------------------------------------------------------      0
UFG4           TTAAATGATCAATTAATTAAAAATGATAACGACATTGATGATGATGCTGAGAAAATTTCA      540
22             ------------------------------------------------------------      0
13             ------------------------------------------------------------      0
19             ------------------------------------------------------------      0
5              ------------------------------------------------------------      0
6              ------------------------------------------------------------      0
```

```
Larissa     TTTTCTACAATCCCATCAAATACTTTTATAGTTTTCGAAAACAGTAGGATTATTGAATCA    600
16          ------------------------------------------------------------    0
10          ------------------------------------------------------------    0
UFG1        TTTTCTACAATCCCATCAAATACTTTTATAGATTTCGAAAACAGTAGGATTATTGAATCA    600
3           ------------------------------------------------------------    0
UF31        TTTTCTACAATCCCATCAAATACTTTTATAGATTTCGAAAACAGTAGGATTATTGAATCA    600
UF33        TTTTCTACAATCCCATCAAATACTTTTATAGATTTCGAAAACAGTAGGATTATTGAATCA    600
PG14T       TTTTCTACAATCCCATCAAATACTTTTATAGATTTCGAAAACAGTAGGATTATTGAATCA    600
23          ------------------------------------------------------------    0
UFG4        TTTTCTACAATCCCATCAAATACTTTTATAGATTTCGAAAACAGTAGGATTATTGAATCA    600
22          ------------------------------------------------------------    0
13          ------------------------------------------------------------    0
19          ------------------------------------------------------------    0
5           ------------------------------------------------------------    0
6           ------------------------------------------------------------    0

Larissa     AATGAAAATTCAGCCACTATTGAAATAGAGTTAAAAACTCAAGATAATATAAAAGAAATT    660
16          ------------------------------------------------------------    0
10          ------------------------------------------------------------    0
UFG1        AATGAAAATTCAGCCACTATTGAAATAGAGTTAAAAACTCAAGATAATATAAAAGAAATT    660
3           ------------------------------------------------------------    0
UF31        AATGAAAATTCAGCCACTATTGAAATAGAGTTAAAAACTCAAGATAATATAAAAGAAATT    660
UF33        AATGAAAATTCAGCCACTATTGAAATAGAGTTAAAAACTCAAGATAATATAAAAGAAATT    660
PG14T       AATGAAAATTCAGCCACTATTGAAATAGAGTTAAAAACTCAAGATAATATAAAAGAAATT    660
23          ------------------------------------------------------------    0
UFG4        AATGAAAATTCAGCCACTATTGAAATAGAGTTAAAAACTCAAGATAATATAAAAGAAATT    660
22          ------------------------------------------------------------    0
13          ------------------------------------------------------------    0
19          ------------------------------------------------------------    0
5           ------------------------------------------------------------    0
6           ------------------------------------------------------------    0

Larissa     TCAAAAAGTGCAGTGTCCCTTTCGTTTGCATACAAAAACAATAATTCTAAAAACTATCTT    720
16          ------------------------------------------------------------    0
10          ------------------------------------------------------------    0
UFG1        TCAAAAAGTGCAGTGTCCCTTTCGTTCGCATACAAAAACAATAATTCTAAAAACTATCTT    720
3           ------------------------------------------------------------    0
UF31        TCAAAAAGTGCAGTATCCCTTTCGTTTGCATACAAAAACAATAATTCTAAAAACTATCTT    720
UF33        TCAAAAAGTGCAGTGTCCCTTTCGTTCGCATACAAAAACAATAATTCTAAAAACTATCTT    720
PG14T       TCAAAAAGTGCAGTATCCCTTTCGTTTGCATACAAAAACAATAATTCTAAAAACTATCTT    720
23          ------------------------------------------------------------    0
UFG4        TCAAAAAGTGCAGTATCCCTTTCGTTTGCATACAAAAACAATAATTCTAAAAACTATCTT    720
22          ------------------------------------------------------------    0
13          ------------------------------------------------------------    0
19          ------------------------------------------------------------    0
5           ------------------------------------------------------------    0
6           ------------------------------------------------------------    0
```

```
Larissa        AATATAACTGGTATTTTAAATAAAAAACAAGACAAAATATTTATAACAGCTAATTTAAAT        780
16             ------------------------------------------------------------        0
10             ------------------------------------------------------------        0
UFG1           AATATAACTGGTATTTTAAATAAAAAACAAGACAAAATATTTATAACAGCTAATTTAAAT        780
3              ------------------------------------------------------------        0
UF31           AATATAACTGGTATTTTAAATAAAAAACAAGACAAAATATTTATAACAGCTAATTTAAAT        780
UF33           AATATAACTGGTATTTTAAATAAAAAACAAGACAAAATATTTATAACAGCTAATTTAAAT        780
PG14T          AATATAACTGGTATTTTAAATAAAAAACAAGACAAAATATTTATAACAGCTAATTTAAAT        780
23             ------------------------------------------------------------        0
UFG4           AATATAACTGGTATTTTAAATAAAAAACAAGACAAAATATTTATAACAGCTAATTTAAAT        780
22             ------------------------------------------------------------        0
13             ------------------------------------------------------------        0
19             ------------------------------------------------------------        0
5              ------------------------------------------------------------        0
6              ------------------------------------------------------------        0


Larissa        AATTTAATACATGGAGAGTCATATTTTTTAGAAAAGGCAAAATTTATTAGTAAGCCAAAA        840
16             ------------------------------------------------------------        0
10             ------------------------------------------------------------        0
UFG1           AATTTAATACATGGGGAGTCATATTTTTTAGAAAAGGCAAAATTTATTAGTAAGCCAAAA        840
3              ------------------------------------------------------------        0
UF31           AATTTAATACATGGGGAGTCATATTTTTTAGAAAAGGCAAAATTTATTAGTAAGCCAAAA        840
UF33           AATTTAATACATGGGGAGTCATATTTTTTAGAAAAAGCAAAATTTATTAGTAAGCCAAAA        840
PG14T          AATTTAATACATGGGGAGTCATATTTTTTAGAAAAGGCAAAATTTATTAGTAAGCCAAAA        840
23             ------------------------------------------------------------        0
UFG4           AATTTAATACATGGGGAGTCATATTTTTTAGAAAAGGCAAAATTTATTAGTAAGCCAAAA        840
22             ------------------------------------------------------------        0
13             ------------------------------------------------------------        0
19             ------------------------------------------------------------        0
5              ------------------------------------------------------------        0
6              ------------------------------------------------------------        0


Larissa        GGATTGTTTTATAACATCAATAATAACTATGAAAACGCTTTTTATGACTTTAACAAATCA        900
16             ------------------------------------------------------------        0
10             ------------------------------------------------------------        0
UFG1           GGATTGTTTTATAACATCAATAATAACTATGAAAACGCTTTTTATGACTTTAACAAATCA        900
3              ------------------------------------------------------------        0
UF31           GGATTGTTTTATAACATCAATAATAACTATGAAAACGCTTTTTATGACTTTAACAAATCA        900
UF33           GGATTGTTTTATAACATCAATAATAACTATGAAAACGCTTTTTATGACTTTAACAAATCA        900
PG14T          GGATTGTTTTATAACATCAATAATAACTATGAAAACGCTTTTTATGACTTTAACAAATCA        900
23             ------------------------------------------------------------        0
UFG4           GGATTGTTTTATAACATCAATAATAACTATGAAAACGCTTTTTATGACTTTTACAAATCA        900
22             ------------------------------------------------------------        0
13             ------------------------------------------------------------        0
19             ------------------------------------------------------------        0
5              ------------------------------------------------------------        0
6              ------------------------------------------------------------        0
```

```
Larissa      AATAATAAAGAACACGAATTCGTAATACACACAGACTCGGAAACATTATTTAAAAATAAC      960
16           ------------------------------------------------------------      0
10           ------------------------------------------------------------      0
UFG1         AATAATAAAGAACATGAATTCGTAATACACACAGATTCAGAAACATTATTTAAAAATAAC      960
3            ------------------------------------------------------------      0
UF31         AATAATAAAGAACACGAATTCGTAATACACACAGATTCAGAAACATTATTTAAAAATAAC      960
UF33         AATAATAAAGAACACGAATTCGTAATACACACAGATTCAGAAACATTGTTTAAAAATAAC      960
PG14T        AATAATAAAGAACACGAATTCGTAATACACACAGATTCAGAAACATTGTTTAAAAATAAC      960
23           ------------------------------------------------------------      0
UFG4         AATAATAAAGAACACGAATTCGTAATACACACAGACTCGGAAACATTATTTAAAAATAAC      960
22           ------------------------------------------------------------      0
13           ------------------------------------------------------------      0
19           ------------------------------------------------------------      0
5            ------------------------------------------------------------      0
6            ------------------------------------------------------------      0

Larissa      TCTAAATTTCAATTTGAGGTTATTGGAGAAATGAAAAACGATCCATTTGAAAATGATCAA      1020
16           ------------------------------------------------------------      0
10           ------------------------------------------------------------      0
UFG1         TCTAAATTTGAATTTGAGGTTATTGGAGAAATGAAAAATGATCCATTTGAAAATGATCAA      1020
3            ------------------------------------------------------------      0
UF31         TCTAAATTTGAATTTGAGGTTATTGGAGAAATGAAAAATGATCCATTTGAAAATGATCAA      1020
UF33         TCTAAATTTGAATTTGAGGTTATTGGTGAAATGAAAAGCGATCCATTTGAAAATGATCAA      1020
PG14T        TCTAAATTTGAATTTGAGGTTATTGGTGAAATGAAAAGCGATCCATTTGAAAATGATCAA      1020
23           ------------------------------------------------------------      0
UFG4         TCTAAATTTGAATTTGAGGTTATTGGAGAAATGAAAAATGATCCATTTGAAAATGATCAA      1020
22           ------------------------------------------------------------      0
13           ------------------------------------------------------------      0
19           ------------------------------------------------------------      0
5            ------------------------------------------------------------      0
6            ------------------------------------------------------------      0

Larissa      AATGAAATTAAATATCACTTAAGTGGTGTTGTTTTAGATAAAAGAAAAATTGATTTTTCT      1080
16           ------------------------------------------------------------      0
10           ------------------------------------------------------------      0
UFG1         AATGAAATTAAATATCACTTAAGTGGTGTTGTTTTAGATAAAAGAAAAATTGATTTTTCT      1080
3            ------------------------------------------------------------      0
UF31         AATGAAATTAAATATCACTTAAGTGGTGTTGTTTTAGATAAAAGAAAAATTGATTTTTCT      1080
UF33         AATGAAATTAAATATCACTTAAGTGGTGTTGTTTTAGATAAAAGAAAAATTGATTTTTCT      1080
PG14T        AATGAAATTAAATATCACTTAAGTGGTGTTGTTTTGGATAAAAGAAAAATTGATTTTTCT      1080
23           ------------------------------------------------------------      0
UFG4         AATGAAATTAAATATCACTTAAGTGGTGTTGTTTTAGATAAAAGAAAAATTGATTTTTCT      1080
22           ------------------------------------------------------------      0
13           ------------------------------------------------------------      0
19           ------------------------------------------------------------      0
5            ------------------------------------------------------------      0
6            ------------------------------------------------------------      0
```

```
Larissa        AAAATTAACGAAAATCTAAGACTAAAATTTTCTAAAGTCGGTAGTGAAGAAACTGATGTT        1140
16             ------------------------------------------------------------        0
10             ------------------------------------------------------------        0
UFG1           AAAATTAATGAAAATCTAAAACTAAAATTTTCTAAAGTCGGTAGTGAAGAAACTGATGTT        1140
3              ------------------------------------------------------------        0
UF31           AAAATTAATGAAAATCTAAAACTAAAATTTTCTAAAGTCGGTAGCGAAGAAACTGATGTT        1140
UF33           AAAATTAATGAAAATCTAAAACTAAAATTTTCTAAAGTCGGTAGCGAAGAAACTGATGTT        1140
PG14T          AAAATTAATGAAAATCTAAAACTAAAATTTTCTAAAGTCGGTAGCGAAGAAACTGATGTT        1140
23             ------------------------------------------------------------        0
UFG4           AAAATTAATGAAAATCTAAAACTAAAATTTTCTAAAGTCGGTAGTGAAGAAACTGATGTT        1140
22             ------------------------------------------------------------        0
13             ------------------------------------------------------------        0
19             ------------------------------------------------------------        0
5              ------------------------------------------------------------        0
6              ------------------------------------------------------------        0


Larissa        TACGCCTCAAAAATAAGTTATGACCCAAATGAAAATAAACTTTCTTTTGAGATTGAAAAT        1200
16             ------------------------------------------------------------        0
10             ------------------------------------------------------------        0
UFG1           TACGCCTCAAAAATAAGTTATGACCCAAATGAAAATAAACTTTCTTTTGAGATAGAAAAT        1200
3              ------------------------------------------------------------        0
UF31           TACGCCTCAAAAATAAGTTATGACCCAAATGAAAATAAACTTTCTTTTGAGATCGAAAAT        1200
UF33           TACGCCTCAAAAATAAGTTATGACCCAAATGAAAATAAACTTTCTTTTGAGATCGAAAAT        1200
PG14T          TACGCCTCAAAAATAAGTTATGACCCAAATGAAAATAAGCTTTCTTTTGAGATAGAAAAT        1200
23             ------------------------------------------------------------        0
UFG4           TACGCCTCAAAAATAAGTTATGACCCAAATGAAAATAAACTTTCTTTTAAGATTGAAAAT        1200
22             ------------------------------------------------------------        0
13             ------------------------------------------------------------        0
19             ------------------------------------------------------------        0
5              ------------------------------------------------------------        0
6              ------------------------------------------------------------        0


Larissa        TCAAATAGTGGTGACCAATTTATATTAAAAGAAATACAAGTAAAAAATAATGAAACAGAA        1260
16             ------------------------------------------------------------        0
10             ------------------------------------------------------------        0
UFG1           TCAAATAGTGGTGACCAATTTATATTAAAAGAAATACAAGTAAAAAATAATGAAACAGAA        1260
3              ------------------------------------------------------------        0
UF31           TCAAATAGTGGTGACCAATTTATATTAAAAGAAATACAAGTAAAAAATAATGAAACAGAA        1260
UF33           TCAAATAGTGGTGACCAATTTATATTAAAAGAAATACAAGTAAAAAATAATGAAACAGAA        1260
PG14T          TCAAATAGTGGTGACCAATTTATATTAAAAGAAATACAAGTAAAAAATAATGAAACAGAA        1260
23             ------------------------------------------------------------        0
UFG4           TCAAATAGTGGTGACCAATTTATATTAAAAGAAATACAAGTAAAAAATAATGAAACAGAA        1260
22             ------------------------------------------------------------        0
13             ------------------------------------------------------------        0
19             ------------------------------------------------------------        0
5              ------------------------------------------------------------        0
6              ------------------------------------------------------------        0
```

```
Larissa     CAGTTTGAGAACTTAGATATAACTAATGTTGATAAAAAACTAATTATTGAATATCCGATT     1320
16          ------------------------------------------------------------     0
10          ------------------------------------------------------------     0
UFG1        CAGTTTGAGAACTTAGATATAACTAATGTTGATAAAAAACTAATTATTGAATATCCGATT     1320
3           ------------------------------------------------------------     0
UF31        CAGTTTGAGAACTTAGATATAACTAATGTTGATAAAAAACTAATTATTGAATATCCGATT     1320
UF33        CAGTTTGAGAACTTAGATATAACTAATGTTGATAAAAAACTAATTATTGAATATCCGATT     1320
PG14T       CAGTTTGAGAACTTAGATATAACTAATGTTGATAAAAAACTAATTATTGAATATCCGATT     1320
23          ------------------------------------------------------------     0
UFG4        CAGTTTGAGAACTTAGATATAACTAATGTTGATAAAAAACTAATTATTGAATATCCGATT     1320
22          ------------------------------------------------------------     0
13          ------------------------------------------------------------     0
19          ------------------------------------------------------------     0
5           ------------------------------------------------------------     0
6           ------------------------------------------------------------     0

Larissa     TCTAAATCGCTTGAAGTTGATTTGATAAATTCAAGATCATGAGAGTCTTCTTTATACCCG     1380
16          ------------------------------------------------------------     0
10          ------------------------------------------------------------     0
UFG1        TCTAAATCGCTTGAAGTTGATTTAATAAATTCAAGATCATGAGAGTCTTCTTTATACCCG     1380
3           ------------------------------------------------------------     0
UF31        TCTAAATCGCTTGAAGTTGATTTGATAAATTCAAGATCATGAGAGTCTTCTTTATACCCG     1380
UF33        TCTAAATCGCTTGAAGTTGATTTGATAAATTCAAGATCATGAGAGTCTTCTTTATACCCG     1380
PG14T       TCTAAATCGCTTGAAGTTGATTTGATAAATTCAAGATCATGAGAGTCTTCTTTATACCCG     1380
23          ------------------------------------------------------------     0
UFG4        TCTAAATCGCTTGAAGTTGATTTGATAAATTCAAGATCATGAGAGTCTTCTTTATACCCA     1380
22          ------------------------------------------------------------     0
13          ------------------------------------------------------------     0
19          ------------------------------------------------------------     0
5           ------------------------------------------------------------     0
6           ------------------------------------------------------------     0

Larissa     AGTTACGTATTTATTAATTTAAAATTAAAAAATAATTGAAATGATGAAAAAATCGAAAAG     1440
16          ------------------------------------------------------------     0
10          ------------------------------------------------------------     0
UFG1        AGTTACGTATTTATTAATTTAAAATTAAAAAATAATTGAAATGATGAAAAAATCGAAAAG     1440
3           ------------------------------------------------------------     0
UF31        AGTTACGTATTTATTAATTTAAAATTAAAAAATAATTGAAATGATGAAAAAATCGAAAAG     1440
UF33        AGTTACGTATTTATTAATTTAAAATTAAAAAATAATTGAAATGATGAAAAAATCGAAAAG     1440
PG14T       AGTTACGTATTTATTAATTTAAAATTAAAAAATAATTGAAATGATGAAAAAATCGAAAAG     1440
23          ------------------------------------------------------------     0
UFG4        AGTTACGTATTTATTAATTTAAAATTAAAAAATAATTGAAATAATGAAAAAATCGAAAAA     1440
22          ------------------------------------------------------------     0
13          ------------------------------------------------------------     0
19          ------------------------------------------------------------     0
5           ------------------------------------------------------------     0
6           ------------------------------------------------------------     0
```

```
Larissa     TTACTTGATCAATTAACGAAAGATGATTATTCGGAATCCAAAAAGAATTCAATAAAGAAT    1500
16          ------------------------------------------------------------    0
10          ------------------------------------------------------------    0
UFG1        TTACTTGATCAATTAACAAAAAATGATTATTCAGAATCCAAAAAGAATTCAATAAAGAAT    1500
3           ------------------------------------------------------------    0
UF31        TTACTTGATCAATTAACGAAAGATGATTATTCGGAATCCAAAAAGAATTCAATAAAGAAT    1500
UF33        TTACTTGATCAATTAACGAAAGATGATTATTCGGAATCCAAAAAGAATTCAATAAAGAAT    1500
PG14T       TTACTTGATCAATTAACGAAAGATGATTATTCGGAATCCAAAAAGAATTCAATAAAGAAT    1500
23          ------------------------------------------------------------    0
UFG4        CTACTTGATCAATTAACAAAAAATGATTATTCGGAATCCAAAAAGAATTCAATAAAGAAT    1500
22          ------------------------------------------------------------    0
13          ------------------------------------------------------------    0
19          ------------------------------------------------------------    0
5           ------------------------------------------------------------    0
6           ------------------------------------------------------------    0

Larissa     GTTCTAGCAAATATTATTTATGATAATTATAAGAAAATAGTTAAGAATAATAATGTTGAT    1560
16          ------------------------------------------------------------    0
10          ------------------------------------------------------------    0
UFG1        GTTCTTGCAAATATTATTTATGACAATTATAAGAAAATAGTTAAAAATAATAACATTGAT    1560
3           ------------------------------------------------------------    0
UF31        GTTCTAGCAAATATTATTTATGATAATTATAAGAAAATAGTTAAAAATAATAATGTTGAT    1560
UF33        GTTCTAGCAAATATTATTTATGATAATTATAAGAAAATAGTTAAGAATAATAATGTTGAT    1560
PG14T       GTTCTAGCAAATATTATTTATGATAATTATAAGAAAATAGTTAAGAATAATAATGTTGAT    1560
23          ------------------------------------------------------------    0
UFG4        ATTCTCGCAAATATTATTTATGACAATTATAAGAAAATAGTTAAAAATAATAACATTGAT    1560
22          ------------------------------------------------------------    0
13          ------------------------------------------------------------    0
19          ------------------------------------------------------------    0
5           ------------------------------------------------------------    0
6           ------------------------------------------------------------    0

Larissa     ATTGATAATAAAATAATAATTTTAAGACTTTCTGACCCAGCTGATGATAGCAACTTTTAT    1620
16          ------------------------------------------------------------    0
10          ------------------------------------------------------------    0
UFG1        ATTGATAATAAAATAATAATTTTAAGACTTTCTGACCCAACTGATGATAGCAACTTTTAT    1620
3           ------------------------------------------------------------    0
UF31        ATTGATAATAAAATAATAATTTTAAGACTTTCTGACCCAGCTGATGATAGCAACTTTTAT    1620
UF33        ATTGATAATAAAATAATAATTTTAAGACTTTCTGACCCAGCTGATGATAGCAACTTTTAT    1620
PG14T       ATTGATAATAAAATAATAATTTTAAGACTTTCTGACCCAGCTGATGATAGCAACTTTTAT    1620
23          ------------------------------------------------------------    0
UFG4        ATTGATAATAAAATAATAATTTTAAGACTTTCTGACCCAACTGATGATAGCAACTTTTAT    1620
22          ------------------------------------------------------------    0
13          ------------------------------------------------------------    0
19          ------------------------------------------------------------    0
5           ------------------------------------------------------------    0
6           ------------------------------------------------------------    0
```

```
Larissa       AGTCAAGATAATAATATTTTTTATTTCCTATTAAATGAAACTAACAAACATTTCAAAAAT      1680
16            ------------------------------------------------------------      0
10            ------------------------------------------------------------      0
UFG1          AGTCAAGATAATAATATTTTTTATTTCCTATTAAATGAAACTAACAAACATTTCAAAAAT      1680
3             ------------------------------------------------------------      0
UF31          AGTCAAGATAATAATATTTTTTATTTCCTATTAAATGAAACTAACAAACATTTCAAAAAT      1680
UF33          AGTCAAGATAATAATATTTTTTATTTCCTATTAAATGAAACTAACAAACATTTCAAAAAT      1680
PG14T         AGTCAAGATAATAATATTTTTTATTTCCTATTAAATGAAACTAACAAACATTTCAAAAAT      1680
23            ------------------------------------------------------------      0
UFG4          AGTCAAGATAATAATATTTTTTATTTCCTATTAAATGAAACTAACAAACATTTCAAAAAT      1680
22            ------------------------------------------------------------      0
13            ------------------------------------------------------------      0
19            ------------------------------------------------------------      0
5             ------------------------------------------------------------      0
6             ------------------------------------------------------------      0

Larissa       ACTAAAGATATTGACGAAAAATGAGATATAGTCAATAATAGCAACACAATTTCTATTTCA      1740
16            ------------------------------------------------------------      0
10            ------------------------------------------------------------      0
UFG1          ACTAAAGATATTGACGAAAAATGAGATATAGTCAATAATAGCAACACAATTTCTATTTCA      1740
3             ------------------------------------------------------------      0
UF31          ACTAAAGATATTGACGAAAAATGAGATATAGTCAATAATAGCAACACAATTTCTATTTCA      1740
UF33          ACTAAAGATATTGACGAAAAATGAGATATAGTCAATAATAGCAATACAATTTCTATTTCA      1740
PG14T         ACTAAAGATATTGACGAAAAATGAGATATAGTCAATAATAGCAACACAATTTCTATTTCA      1740
23            ------------------------------------------------------------      0
UFG4          ACTAAAGATATTGACGAAAAATGAGATATAGTCAATAATAGCAACACAATTTCTATTTCA      1740
22            ------------------------------------------------------------      0
13            ------------------------------------------------------------      0
19            ------------------------------------------------------------      0
5             ------------------------------------------------------------      0
6             ------------------------------------------------------------      0

Larissa       ATGATTAAAAATCCATTTATAAGCAACAACAGAAATGGAACAGCAATTGAAAATGATCAC      1800
16            ------------------------------------------------------------      0
10            -------------------------------------------CAATTGAAAATGATCAC      17
UFG1          ATGATTAAAAATCCATTTATAAGCAACAACAGAAATGGAACAGCTATTGAAAATGATCAC      1800
3             ------------------------------------------------------------      0
UF31          ATGATTAAAAATCCATTTATAAGCAACAACAGAAATGGAACAGCAATTGAAAATGATCAC      1800
UF33          ATGATTAAAAATCCATTTATAAGCAACAACAGAAATGGAACAGCAATTGAAAATGATCAC      1800
PG14T         ATGATTAAAAATCCATTTATAAGCAACAACAGAAATGGAACAGCAATTGAAAATGATCAC      1800
23            ------------------------------------------------------------      0
UFG4          ATGATTAAAAATCCATTTATAAGCAACAACAGAAATGGAACAGCTATTGAAAATGATCAC      1800
22            ------------------------------------GGAACAGCAATTGAAAATGATCAC      24
13            ------------------------------------------------------------      0
19            ------------------------------------------------------------      0
5             ------------------------------------------------------------      0
6             ------------------------------------------------------------      0
```

```
Larissa      TTTAAAATAAAAAGACTAAGTAGCGAAACATTATTTGCTCACAACGAAGACGGATCACAC      1860
16           ------------------------------------------------------------      0
10           TTTAAAATAAAAAGACTAAGTAGCGAAACATTATTTGCTCACAACGAAGACGGATCACAC      77
UFG1         TTTAAAATAAAAAGACTAAGTAGCGAAACATTATTTGCTCACAACGAAGACGGATCGCAC      1860
3            -------------------GTAGCGAAACATTATTTGCTCACAACGAAGACGGATCACAC      41
UF31         TTTAAAATAAAAAGACTAAGTAGCGAAACATTATTTGCTCACAACGAAGACGGATCACAC      1860
UF33         TTTAAAATAAAAAGACTAAGTAGCGAAACATTATTTGCTCACAACGAAGACGGATCACAC      1860
PG14T        TTTAAAATAAAAAGACTAAGTAGCGAAACATTATTTGCTCACAACGAAGACGGATCACAC      1860
23           --------------------TAGCGAGACATTATTTGCTCACAACGAAGACGGATCACAC      40
UFG4         TTTAAAATAAAAAGACTAAGTAGCGAAACATTATTTGCTCACAACGAAGACGGATCACAC      1860
22           TTTAAAATAAAAAGACTAAGTAGCGAAACATTATTTGCTCACAACGAAGACGGATCACAC      84
13           -------------------GTAGCGAAACATTATTTGCTCACAACGAAGACGGATCACAC      41
19           --------------------TAGCGAAACATTATTTGCTCACAACGAAGACGGATCACAC      40
5            --------------------TAGCGAAACATTATTTGCTCACAACGAAGACGGATCACAC      40
6            -------------------GTAGCGAAACATTATTTGCTCACAACGAAGACGGATCACAC      41
```

RIP motif

```
Larissa      TCATATCGTATCCCAAATGTTACTAAACTTAAAAACGGAAAAATCCTTAGCGTTGTGGAT      1920
16           ------------------------------------------------------------      0
10           TCATATCGTATCCCAAATGTTACTAAACTTAAAAACGGAAAAATCCTTAGCGTTGTGGAT      137
UFG1         TCATATCGTATCCCAAATGTTACTAAACTTAAAAACGGAAAAATCCTTAGCGTTGTGGAC      1920
3            TCATATCGTATCCCAAATGTTACTAAACTTAAAAACGGAAAAATCCTTAGCGTTGTTGAT      101
UF31         TCATATCGTATCCCAAATGTTACTAAACTTAAAAACGGAAAAATCCTTAGCGTTGTGGAT      1920
UF33         TCATATCGTATCCCAAATGTTACTAAACTTAAAAACGGAAAAATCCTTAGCGTTGTGGAT      1920
PG14T        TCATATCGTATCCCAAATGTTACTAAACTTAAAAACGGAAAAATCCTTAGCGTTGTGGAT      1920
23           TCATATCGTATCCCAAATGTTACTAAACTTAAAAACGGAAAAATCCTTAGCGTTGTAGAT      100
UFG4         TCATATCGTATCCCAAATGTTACTAAACTTAAAAACGGAAAAATCCTTAGCGTTGTGGAT      1920
22           TCATATCGTATCCCAAATGTTACTAAACTTAAAAACGGAAAAATCCTTAGCGTTGTGGAT      144
13           TCATATCGTATCCCAAATGTTACTAAACTTAAAAACGGAAAAATCCTTAGCGTTGTGGAT      101
19           TCATATCGTATCCCAAATGTTACTAAACTTAAAAACGGAAAAATCCTTAGCGTTGTGGAT      100
5            TCATATCGTATCCCAAATGTTACTAAACTTAAAAACGGAAAAATCCTTAGCGTTGTGGAT      100
6            TCATATCGTATCCCAAATGTTACTAAACTTAAAAACGGAAAAATCCTTAGCGTTGTGGAT      101


Larissa      AAAAGAGCAGAAAACATTAGTGACTATAATAACTCAATTTCACAAGTTTTTAAAGAATCA      1980
16           ------------------------------------------------------------      0
10           AAAAGAGTAGAAAACATTAGTGACTATAATAACTCAATTTCACAAGTTTTTAAAGAATCA      197
UFG1         AAAAGAGTAGAAAACATTAGTGACTATAATAACTCAATTTCACAAGTTTTTAAAGAATCA      1980
3            AAAAGAGTAGAAAACATTAGTGACTATAATAACTCAATTTCACAAGTTTTTAAAGAATCA      161
UF31         AAAAGAGTAGAAAACATTAGTGACTATAATAACTCAATTTCACAAGTTTTTAAAGAAACA      1980
UF33         AAAAGAGTAGAAAACATTAGTGACTATAATAACTCAATTTCACAAGTTTTTAAAGAATCA      1980
PG14T        AAAAGAGTAGAAAACATTAGTGACTATAATAACTCAATTTCACAAGTTTTTAAAGAATCA      1980
23           AAAAGAGTAGAAAACATTAGTGACTATAATAACTCAATTTCACAAGTTTTTAAAGAATCA      160
UFG4         AAAAGAGTAGAAAACATTAGTGACTATAATAACTCAATTTCACAAGTTTTTAAAGAATCA      1980
22           AAAAGAGTAGAAAACATTAGTGACTATAATAACTCAATTTCACAAGTTTTTAAAGAATCA      204
13           AAAAGAGTAGAAAACATTAGTGACTATAATAACTCAATTTCACAAGTTTTTAAAGAATCA      161
19           AAAAGAGTAGAAAACATTAGTGACTATAATAACTCAATTTCACAAGTTTTTAAAGAATCA      160
5            AAAAGAGTAGAAAATATTAGTGACTATAATAACTCAATTTCACAAGTTTTTAAAGAATCA      160
6            AAAAGAGTAGAAAATATTAGTGACTATAATAACTCAATTTCACAAGTTTTTAAAGAATCA      161
```

```
Larissa    TTAGATGGTGGAAAAACTTGAAGCCAAAATAAAGAAATCTTAAAAATAGCTGTTCCGAAG    2040
16         ------------------------------------------------------------    0
10         TTAGATGGTGGAAAAACTTGAAGTCAAAATAAAGAAATCTTAAAAATAGCTGTTCCAAAG    257
UFG1       TTAGATGGCGGAAAAACTTGAAGCCAAAATAAAGAAATCTTAAAAATAGCTGTTCCAAAG    2040
3          TTAGATGGCGGAAAAACTTGAAGCCAAAATAAAGAAATCTTAAAAATAGCTGTTCCGAAG    221
UF31       TTAGATGGCGGAAAAACTTGAAGCCAAAATAAAGAAATCTTAAAAATAGCTGTTCCAAAG    2040
UF33       TTAGATGGCGGAAAAACTTGAAGCCAAAATAAAGAAATCTTAAAAATAGCTGTTCCAAAG    2040
PG14T      TTAGATGGCGGAAAAACTTGAAGCCAAAATAAAGAAATCTTAAAAATAGCTGTTCCAAAG    2040
23         TTAGATGGCGGAAAAACTTGAAGCCAAAATAAAGAAATCTTAAAAATAGCTGTTCCAAAG    220
UFG4       TTAGATGGCGGAAAAACTTGAAGCCAAAATAAAGAAATCTTAAAAATAGCTGTTCCAAAG    2040
22         TTAGATGGCGGAAAAACTTGAAGCCAAAATAAAGAAATCTTAAAAATAGCTGTTCCAAAG    264
13         TTAGATGGCGGAAAAACTTGAAGCCAAAATAAAGAAATCTTAAAAATAGCTGTTCCAAAG    221
19         TTAGATGGCGGAAAAACTTGAAGCCAAAATAAAGAAATCTTAAAAATAGCTGTTCCAAAG    220
5          TTAGATGGCGGAAAAACTTGAAGCCAAAATAAAGAAATCTTAAAAATAGCTGTTCCAAAG    220
6          TTAGATGGCGGAAAAACTTGAAGCCAAAATAAAGAAATCTTAAAAATAGCTGTTCCAAAG    221

Larissa    AAAAACAATCGTGGTATAGCTATAGATGGTATAATAACAGAAGTTGAGTATTTTGATGAA    2100
16         ------------------------------------------------------------    0
10         AAAAACAATCGTGGTATAGCTATAGATGGTATAATAACAGAAATTGAGTATTTTGATGAA    317
UFG1       AAAAACAATCGTGGTATAGCTATAGATGGTATAATAACAGAAATTGAGTATTTTGATGAA    2100
3          AAAAACAATCGCGGTATAGCTATAGACGGTATAATAACAGAAGTTGAGTATTTTGATGAA    281
UF31       AAAAACAATCGTGGTATAGCTATAGATGGTATAATAACAGAAATTGAGTATTTTGATGAA    2100
UF33       AAAAACAATCGTGGTATAGCTATAGATGGTATAATAACAGAAATTGAGTATTTTGATGAA    2100
PG14T      AAAAACAATCGTGGTATAGCTATAGATGGTATAATAACAGAAATTGAGTATTTTGATGAA    2100
23         AAAAACAATCGTGGTATAGCTATAGATGGTATAATAACAGAAATTGAGTATTTTGATGAA    280
UFG4       AAAAACAATCGTGGTATAGCTATAGATGGTATAATAACAGAAATTGAGTATTTTGATGAA    2100
22         AAAAACAATCGTGGTATAGCTATAGATGGTATAATAACAGAAATTGAGTATTTTGATGAA    324
13         AAAAACAATCGTGGTATAGCTATAGATGGTATAATAACAGAAATTGAGTATTTTGATGAA    281
19         AAAAACAATCGTGGTATAGCTATAGATGGTATAATAACAGAAATTGAGTATTTTGATGAA    280
5          AAAAACAATCGTGGTATAGCTATAGATGGTATAATAACAGAAATTGAGTATTTTGATGAA    280
6          AAAAACAATCGCGGTATAGCTATAGATGGTATAATAACAGAAGTTGAGTATTTTGATGAA    281

Larissa    GAAACACAAACTAATAAAACAAAGCTTCATTTTATTGTTGATATTTTTCCAGGAACAAAT    2160
16         ------------------------------------------------------------    0
10         GAAACACAAACTAATAAAACAAAGCTTCATTTTATTGTTGATATTTTTCCAGGAACAAAT    377
UFG1       GAAACACAAACTAATAAAACAAAGCTTCATTTTATTGTTGATATTTTTCCAGGAACAAAT    2160
3          GAAACACAAACTAATAAAACAAAGCTTCATTTTATTGTTGATATTTTTCCAGGAACAAAT    341
UF31       GAAACACAAACTAATAAAACAAAGCTTCATTTTATTGTTGATATTTTTCCAGGAACAAAT    2160
UF33       GAAACACAAACTAATAAAACAAAGCTTCATTTTATTGTTGATATTTTTCCAGGAACAAAT    2160
PG14T      GAAACACAAACTAATAAAACAAAGCTTCATTTTATTGTTGATATTTTTCCAGGAACAAAT    2160
23         GAAACACAAACTAATAAAACAAAGCTTCATTTTATTGTTGATATTTTTCCAGGAACAAAT    340
UFG4       GAAACACAAACTAATAAAACAAAGCTTCATTTTATTGTTGATATTTTTCCAGGAACAAAT    2160
22         GAAACACAAACTAATAAAACAAAGCTTCATTTTATTGTTGATATTTTTCCAGGAACAAAT    384
13         GAAACACAAACTAATAAAACAAAGCTTCATTTTATTGTTGATATTTTTCCAGGAACAAAT    341
19         GAAACACAAACTAATAAAACAAAGCTTCATTTTATTGTTGATATTTTTCCAGGAACAAAT    340
5          GAAACACAAACTAATAAAACAAAGCTTCATTTTATTGTTGATATTTTTCCAGGAACAAAT    340
6          GAAACACAAACTAATAAAACAAAGCTTCATTTTATTGTTGATATTTTTCCAGGAACAAAT    341
```

```
Larissa      ACTGGTGTTCCACATTTATCATCGGGTAATCCATGATTCTATATAGGTGATCAAGGTTAT      2220
16           --------------------------------------------------------TTAT      4
10           ACTGGTGTTCCACATTTATCATCGGGTAACCCATGATTCTATATAGGTGATCAAGGTTAT      437
UFG1         ACTGGTGTTCCACATTTATCATCAGGTAACCCATGATTCTATATAGGTGATCAAGGTTAT      2220
3            ACTGGTGTTCCACATTTATCATCGGGTAACCCATGATTCTATATAGGTGATCAAGGTTAT      401
UF31         ACTGGTGTTCCACATTTATCATCGGGTAACCCATGATTCTATATAGGTGATCAAGGTTAT      2220
UF33         ACTGGTGTTCCACATTTATCATCGGGTAACCCATGATTCTATATAGGTGATCAAGGTTAT      2220
PG14T        ACTGGTGTTCCACATTTATCATCGGGTAACCCATGATTCTATATAGGTGATCAAGGTTAT      2220
23           ACTGGTGTTCCACATTTATCATCGGGTAACCCATGATTCTATATAGGTGATCAAGGTTAT      400
UFG4         ACTGGTGTTCCACATTTATCATCGGGTAACCCATGATTCTATATAGGTGATCAAGGTTAT      2220
22           ACTGGTGTTCCACATTTATCATCGGGTAACCCATGATTCTATATAGGTGATCAAGGTTAT      444
13           ACTGGTGTTCCACATTTATCATCGGGTAACCCATGATTCTATATAGGTGATCAAGGTTAT      401
19           ACTGGTGTTCCACATTTATCATCGGGTAACCCATGATTCTATATAGGTGATCAAGGTTAT      400
5            ACTGGTGTTCCACATTTATCATCGGGTAACCCATGATTCTATATAGGTGATCAAGGTTAT      400
6            ACTGGTGTTCCACATTTATCATCGGGTAACCCATGATTCTATATAGGTGATCAAGGTTAT      401
                                                                     ****

Larissa      CTAAAAATGTGGACAAAATTAAATAACAGAAATAATTTTGATTCTAGATCATCAGTATTA      2280
16           CTAAAAATGTGGACAAAATTAAATAACAGAAATAATTTTGATTGTAGATCATCAGTATTA      64
10           CTAAAAATGTGGACAAAATTAAATAACAGAAATAATTTTGATTCCAGATCATCAGTATTA      497
UFG1         CTAAAAATGTGGACAAAATTAAATAACAGAAATAATTTTGATTCTAGATCATCAGTATTA      2280
3            CTAAAAATGTGGACAAAATTAAATAACAGAAATAATTTTGATTCTAGATCATCAGTATTA      461
UF31         CTAAAAATGTGGACAAAATTAAATAACAGAAATAATTTTGATTCTAGATCATCAGTATTA      2280
UF33         CTAAAAATGTGGACAAAATTAAATAACAGAAATAATTTTGATTCTAGATCATCAGTATTA      2280
PG14T        CTAAAAATGTGGACAAAATTAAATAACAGAAATAATTTTGATTCTAGATCATCAGTATTA      2280
23           CTAAAAATGTGGACAAAATTAAATAACAGAAATAATTTTGATTCTAGATCATCAGTATTA      460
UFG4         CTAAAAATGTGGACAAAATTAAATAACAGAAATAATTTTGATTCTAGATCATCAGTATTA      2280
22           CTAAAAATGTGGACAAAATTAAATAACAGAAATAATTTTGATTCTAGATCATCAGTATTA      504
13           CTAAAAATGTGGACAAAATTAAATAACAGAAATAATTTTGATTCTAGATCATCAGTATTA      461
19           CTAAAAATGTGGACAAAATTAAATAACAGAAATAATTTTGATTCTAGATCATCAGTATTA      460
5            CTAAAAATGTGGACAAAATTAAATAACAGAAATAATTTTGATTCTAGATCATCAGTATTA      460
6            CTAAAAATGTGGACAAAATTAAATAACAGAAATAATTTTGATTCTAGATCATCAGTATTA      461
             *******************************************  ***************

Larissa      AAGCGTGTAGAAGGAAGAGGAAATTGATTTAGAAGATATATACTACCAGCTGGTGTTTCA      2340
16           AAGCGTATAGAAGGAAGAGGAAATTGATTTAGAAGATATATACTACCAGCTGGTGTTTCA      124
10           AAGCGTGTAGAAGGAAGAGGAAATTGATTTAGAAGATATATACTACCAGCTGGTGTTTCA      557
UFG1         AAGCGTGTAGAAGGAAGAGGAAATTGATTTAGAAGATATATACTACCAGCTGGTGTTTCA      2340
3            AAGCGTGTAGAAGGAAGAGGAAATTGATTTAGAAGATATATACTACCAGCTGGTGTTTCA      521
UF31         AAGCGTGTAGAAGGAAGAGGAAATTGATTTAGAAGATATATACTACCAGCTGGTGTTTCA      2340
UF33         AAGCGTGTAGAAGGAAGAGGAAATTGATTTAGAAGATATATACTACCAGCTGGTGTTTCA      2340
PG14T        AAGCGTGTAGAAGGAAGAGGAAATTGATTTAGAAGATATATACTACCAGCTGGTGTTTCA      2340
23           AAGCGTGTAGAAGGAAGAGGAAATTGATTTAGAAGATATATACTACCAGCTGGTGTTTCA      520
UFG4         AAGCGTGTAGAAGGAAGAGGAAATTGATTTAGAAGATATATACTACCAGCTGGTGTTTCA      2340
22           AAGCGTGTAGAAGGAAGAGGAAATTGATTTAGAAGATATATACTACCAGCTGGTGTTTCA      564
13           AAGCGTGTAGAAGGAAGAGGAAATTGATTTAGAAGATATATACTACCAGCTGGTGTTTCA      521
19           AAGCGTGTAGAAGGAAGAGGAAATTGATTTAGAAGATATATACTACCAGCTGGTGTTTCA      520
5            AAGCGTGTAGAAGGAAGAGGAAATTGATTTAGAAGATATATACTACCAGCTGGTGTTTCA      520
6            AAGCGTGTAGAAGGAAGAGGAAATTGATTTAGAAGATATATACTACCAGCTGGTGTTTCA      521
             ****** *****************************************************
```

```
Larissa        TTTAACAATAATTTCACAGCAAGCACACAACTAGAAGAAACAAATACATATGTGGATATG     2400
16             TTTAACAATAATTTCACAGCAAGCACACAACTAGAAGAAACAAATACATATGTGGATATG     184
10             TTTAACAATAATTTCACAGCAAGCACACAACTAGAAGAAACAAATACATATGTGGATATG     617
UFG1           TTTAACAATAATTTCACAGCAAGCACACAACTAGAAGAAACAAATACATATGTGGATATG     2400
3              TTTAACAATAATTTCACAGCAAGCACACAACTAGAAGAAACAAATACATATGTGGATATG     581
UF31           TTTAACAATAATTTCACAGCAAGCACACAACTAGAAGAAACAAATACATATGTGGATATG     2400
UF33           TTTAACAATAATTTCACAGCAAGCACACAACTAGAAGAAACAAATACATATGTGGATATG     2400
PG14T          TTTAACAATAATTTCACAGCAAGCACACAACTAGAAGAAACAAATACATATGTGGATATG     2400
23             TTTAACAATAATTTCACAGCAAGCACACAACTAGAAGAAACAAATACATATGTGGATATG     580
UFG4           TTTAACAATAATTTCACAGCAAGCACACAACTAGAAGAAACAAATACATATGTGGATATG     2400
22             TTTAACAATAATTTCACAGCAAGCACACAACTAGAAGAAACAAATACATATGTGGATATG     624
13             TTTAACAATAATTTCACAGCAAGCACACAACTAGAAGAAACAAATACATATGTGGATATG     581
19             TTTAACAATAATTTCACAGCAAGCACACAACTAGAAGAAACAAATACATATGTGGATATG     580
5              TTTAACAATAATTTCACAGCAAGCACACAACTAGAAGAAACAAATACATATGTGGATATG     580
6              TTTAACAATAATTTCACAGCAAGCACACAACTAGAAGAAACAAATACATATGTGGATATG     581
               ************************************************************

Larissa        AATTATCATCAAGATACAAAATCAATTTCTGGTAGAGTATATGAAAATGTCATGGAATCA     2460
16             AATTATCATCAAGATACAAAATCAATTTCTGGTAGAGTATATGAAAATGTCATGGAATCA     244
10             AATTATCATCAAGATACAAAATCAATTTCTGGTAGAGTATATGAAAATGTCATGGAATCA     677
UFG1           AATTATCATCAAGATACAAAATCAATTTCTGGTAGAGTATATGAAAATGTCATGGAATCA     2460
3              AATTATCATCAAGATACAAAATCAATTTCTGGTAGAGTATATGAAAATGTCATGGAATCA     641
UF31           AATTATCATCAAGATACAAAATCAATTTCTGGTAGAGTATATGAAAATGTCATGGAATCA     2460
UF33           AATTATCATCAAGATACAAAAACAATTTCTGGTAGAGTATATGAAAATGTCATGGAATCA     2460
PG14T          AATTATCATCAAGATACAAAATCAATTTCTGGTAGAGTATATGAAAATGTCATGGAATCA     2460
23             AATTATCATCAAGATACAAAATCAATTTCTGGTAGAGTATATGAAAATGTCATGGAATCA     640
UFG4           AATTATCATCAAGATACAAAATCAATTTCTGGTAGAGTATATGAAAATGTCATGGAATCA     2460
22             AATTATCATCAAGATACAAAATCAATTTCTGGTAGAGTATATGAAAATGTCATGGAATCA     684
13             AATTATCATCAAGATACAAAATCAATTTCTGGTAGAGTATATGAAAATGTCATGGAATCA     641
19             AATTATCATCAAGATACAAAATCAATTTCTGGTAGAGTATATGAAAATGTCATGGAATCA     640
5              AATTATCATCAAGATACAAAATCAATTTCTGGTAGAGTATATGAAAATGTCATGGAATCA     640
6              AATTATCATCAAGATACAAAATCAATTTCTGGTAGAGTATATGAAAATGTCATGGAATCA     641
               ********************* **************************************

Larissa        GATTTTGATGATCCAGCAGCATTAGATTCTAAAAAAACTGAGCACAGTGTTTTTGATGAA     2520
16             GATTTTGATGATCCAGCGGCATTAGATTCTAAAAAAACTGAGCACAGTGTTTTTGATGAA     304
10             GATTTTGATGATCCAGCGGCATTAGATTCTAAAAAAACTGAGCACAGTGTTTTTGATGAA     737
UFG1           GATTTTGATGATCCAGCGGCATTAGATTCTAAAAAAACTGAGCACAGTGTTTTTGATGAA     2520
3              GATTTTGATGATCCAGCGGCATTAGATTCTAAAAAAACTGAGCACAGTGTTTTTGATGAA     701
UF31           GATTTTGATGATCCAGCGGCATTAGATTCTAAAAAAACTGAGCACAGTGTTTTTGATGAA     2520
UF33           GATTTTGATGATCCAGCGGCATTAGATTCTAAAAAAACTGAGCACAGTGTTTTTGATGAA     2520
PG14T          GATTTTGATGATCCAATGGCATTAGATTCTAAAAAAACTGAGCACAGTGTTTTTGATGAA     2520
23             GATTTTGATGATCCAGCGGCATTAGATTCTAAAAAAACTGAGCACAGTGTTTTTGATGAA     700
UFG4           GATTTTGATGATCCAGCGGCATTAGATTCTAAAAAAACTGAGCACAGTGTTTTTGATGAA     2520
22             GATTTTGATGATCCAGCGGCATTAGATTCTAAAAAAACTGAGCACAGTGTTTTTGATGAA     744
13             GATTTTGATGATCCAGCGGCATTAGATTCTAAAAAAACTGAGCACAGTGTTTTTGATGAA     701
19             GATTTTGATGATCCAGCGGCATTAGATTCTAAAAAAACTGAGCACAGTGTTTTTGATGAA     700
5              GATTTTGATGATCCAGCGGCATTAGATTCTAAAAAAACTGAGCACAGTGTTTTTGATGAA     700
6              GATTTTGATGATCCAGCGGCATTAGATTCTAAAAAAACTGAGCACAGTGTTTTTGATGAA     701
               ***************   ******************************************
```

```
Larissa     CCAAGAAAAGTTACAAACGTAAACAATAATACATTTGAACCATTAAGAAATGAGCATGCT   2580
16          CCAAGAAAAGTTACAAACGTAAACAATAATACATTTGAACCATTAAGAAATGAGCATGCT   364
10          CCAAGAAAAGTTACAAACGTAAACAATAATACATTTGAACCACTAAGAAATGAGCACGCT   797
UFG1        CCAAGAAAAGTTACAAACGTAAACAATAATACATTTGAACCATTAAGAAATGAGCATGCT   2580
3           CCAAGAAAAGTTACAAACGTAAACAATAATACATTTGAACCATTAAGAAATGAGCATGCT   761
UF31        CCAAGAAAAGTTACAAACGTAAACAATAATACATTTGAACCATTAAGAAATGAGCATGCT   2580
UF33        CCAAGAAAAGTTACAAACGTAAACAATAATACATTTGAACCATTAAGAAATGAGCATGCT   2580
PG14T       CCAAGAAAAGTTACAAACGTAAACAATAATACATTTGAACCATTAAGAAATGAGCATGCT   2580
23          CCAAGAAAAGTTACAAACGTAAACAATAATACATTTGAACCATTAAGAAATGAGCACGCT   760
UFG4        CCAAGAAAAGTTACAAACGTAAACAATAATACATTTGAACCATTAAGAAATGAGCATGCT   2580
22          CCAAGAAAAGTTACAAACGTAAACAATAATACATTTGAACCATTAAGAAATGAGCATGCT   804
13          CCAAGAAAAGTTACAAACGTAAACAATAATACATTTGAACCATTAAGAAATGAGCATGCT   761
19          CCAAGAAAAGTTACAAACGTAAACAATAATACATTTGAACCATTAAGAAATGAGCATGCT   760
5           CCAAGAAAAGTTACAAACGTAAACAATAATACATTTGAACCATTAAGAAATGAGCATGCT   760
6           CCAAGAAAAGTTACAAACGTAAACAATAATACATTTGAACCATTAAGAAATGAGCATGCT   761
            ******************************************* ************ ***
                                                                          Asp box motif
Larissa     GTATATGCCTTAGCCATAAATAGTCACCTTGCGACGCTAGAAAGTTATGATGAAGGTAGA   2640
16          GTATATGCCTTGGCCATAAATAGTCACCTTGCAACGCTAGAAAGTTATGATGAAGGTAGA   424
10          GTATATGCCTTGGCCATAAATAGCCACCTTGCAACGCTAGAAAGTTATGATGAAGGTAGA   857
UFG1        GTATATGCCTTAGCCATAAATAGCCACCTTGCGACGCTAGAAAGTTATGATGAAGGTAGA   2640
3           GTATATGCCTTGGCCATAAATAGTCACCTTGCAACGCTAGAAAGTTATGATGAAGGTAGA   821
UF31        GTATATGCCTTAGCCATAAATAGCCACCTTGCGACGCTAGAAAGTTATGATGAAGGTAGA   2640
UF33        GTATATGCCTTAGCCATAAATAGCCACCTTGCGACGCTAGAAAGTTATGATGAAGGTAGA   2640
PG14T       GTATATGCCTTAGCCATAAATAGCCACCTTGCAACACTAGAAAGTTATGATGAAGGTAGA   2640
23          GTATATGCCTTGGCCATAAATAGTCACCTTGCAACGCTAGAAAGTTATGATGAAGGTAGA   820
UFG4        GTATATGCCTTGGCCATAAATAGTCACCTTGCAACGCTAGAAAGTTATGATGAAGGTAGA   2640
22          GTATATGCCTTGGCCATAAATAGTCACCTTGCAACGCTAGAAAGTTATGATGAAGGTAGA   864
13          GTATATGCCTTGGCCATAAATAGTCACCTTGCAACGCTAGAAAGTTATGATGAAGGTAGA   821
19          GTATATGCCTTGGCCATAAATAGTCACCTTGCAACGCTAGAAAGTTATGATGAAGGTAGA   820
5           GTATATGCCTTGGCCATAAATAGTCACCTTGCAACGCTAGAAAGTTATGATGAAGGTAGA   820
6           GTATATGCCTTGGCCATAAATAGTCACCTTGCAACGCTAGAAAGTTATGATGAAGGTAGA   821
            *********** *********** ******** ** ************************

Larissa     ACATGAACTAACTTACAATGGATCGATGAAAAACTATCAAGACATAGAAATAATCATAAG   2700
16          ACATGAACTAACTTAAAATGGATTGATGAAAAACTCTCAAGAGATAG-------------   471
10          ACATGAACTAACTTACAATGAATCGATGAAAAACTCTCAAGACATAG-------------   904
UFG1        ACATGAACTAACTTACAATGGATCGATGAAAAACTATCAAGACATAGAAATAATCATAAG   2700
3           ACATGAACTAACTTACAATGGATTGATGAAAAACTCTCAAGACAT---------------   866
UF31        ACATGAACTAACTTACAATGGATCGATGAAAAACTATCAAAACATAGAAATAATCATAAG   2700
UF33        ACATGAACTAACTTACAATGGATCGATGAAAAACTATCAAAACATAGAAATAATCATAAG   2700
PG14T       ACATGAACTAACTTACAATGGATTGATGAAAAACTTTCAAGACATAGAAATAATCATAAG   2700
23          ACATGAACTAACTTACAATGGATTGATGAAAAACTTTCAAGAGATAG-------------   867
UFG4        ACATGAACTAACTTACAATGGATCGATGAAAAACTCTCAAGAGATAGAAATAATCATAAG   2700
22          ACATGAACTAACTTACAATGGATCGATGAAAAACTCTCAAGAGATAGAAATAATCATAAG   924
13          ACATGAACTAACTTACAATGGATCGATGAAAAACTCTCAAGACAT---------------   866
19          ACATGAACTAACTTACAATGGATCGATGAAAAACTCTCAAGAC-----------------   863
5           ACATGAACTAACTTACAATGGATTGATGAAAAACTTTCAAGACATAG-------------   867
6           ACATGAACTAACTTACAATGGATTGACGAAAAACTTTCAAGACATAGAAATAATC-----   876
            *************** **** ** ** ******** **** *
```

```
Larissa     TTTGTAGGTACTGGGGTAGGAAACGGAATACAACTTAAACACCAAGCAAATGCTTCTATA     2760
16          ------------------------------------------------------------     471
10          ------------------------------------------------------------     904
UFG1        TTTGTAGGTACTGGGGTAGGAAACGGAATACAACTTAAACACCAAACAAATGCTTCTATA     2760
3           ------------------------------------------------------------     866
UF31        TTTGTAGGTACTGGGGTAGGAAACGGAATACAACTTAAACACCAAGCAAATGCTTCTATA     2760
UF33        TTTGTAGGTACTGGGGTAGGAAACGGAATACAACTTAAACACCAAACAAATGCTTCTATA     2760
PG14T       TTTGTAGGTACTGGGGTAGGAAACGGAATACAACTTAAACACCAAGCAAATGCTTCTATA     2760
23          ------------------------------------------------------------     867
UFG4        TTTGTAGGTACTGGGGTAGGAAACGGAATACAACTTAAACACCAAGCAAATGCTTCTATA     2760
22          TTTGTAGGTACTGGGGTAGGAAACGGAATACAA---------------------------     957
13          ------------------------------------------------------------     866
19          ------------------------------------------------------------     863
5           ------------------------------------------------------------     867
6           ------------------------------------------------------------     876


Larissa     AATGGTAGAGTAATTATACCTATGTACTCTATGAATAACAATGATCATTATATGTTTTTC     2820
16          ------------------------------------------------------------     471
10          ------------------------------------------------------------     904
UFG1        AATGGTAGAGTAATTATACCTATGTACTCTATGAATAACAATGATCACCATATGTTTTTC     2820
3           ------------------------------------------------------------     866
UF31        AATGGTAGAGTAATTATACCTATGTACTCTATGAATAACAATGATCATTATATGTTTTTC     2820
UF33        AATGGTAGAGTAATTATACCTATGTACTCTATGAATAACAATGATCATTATATGTTTTTC     2820
PG14T       AATGGTAGAGTAATTATACCTATGTACTCTATGAATAACAATGATCATTATATGTTTTTC     2820
23          ------------------------------------------------------------     867
UFG4        AATGGTAGAGTAATTATACCTATGTACTCTATGAATAACAATGATCATTATATGTTTTTC     2820
22          ------------------------------------------------------------     957
13          ------------------------------------------------------------     866
19          ------------------------------------------------------------     863
5           ------------------------------------------------------------     867
6           ------------------------------------------------------------     876


Larissa     ATATATAGCGATGATAAAGGAAAAACATGAACCAAATACACACCTAATGGATTTAAAACC     2880
16          ------------------------------------------------------------     471
10          ------------------------------------------------------------     904
UFG1        ATATACAGCGATGATAAAGGGAAAACATGAACCAAATACACGCCTACCGGATTTAAAGCC     2880
3           ------------------------------------------------------------     866
UF31        ATATATAGCGATGATAAAGGAAAAACATGAACCAAATACACACCTACTGGATTTAAAACC     2880
UF33        ATATATAGCGATGATAAAGGAAAAATATGAACCAAATACACACCTACTGGATTTAAAACT     2880
PG14T       ATATATAGCGATGATAAAGGAAAAACATGAACCAAATACACACCTAATGGATTTAAAACC     2880
23          ------------------------------------------------------------     867
UFG4        ATATATAGCGATGATAAAGGAAAAACATGAATCAAATACACACCTACTGGATTTAAAACT     2880
22          ------------------------------------------------------------     957
13          ------------------------------------------------------------     866
19          ------------------------------------------------------------     863
5           ------------------------------------------------------------     867
6           ------------------------------------------------------------     876
```

```
Larissa     AACCTATCTGAATCATCTTTTGTTGAAACAGAAGACGGTACTTTATATTGATTCGCAAGA     2940
16          ------------------------------------------------------------     471
10          ------------------------------------------------------------     904
UFG1        AACTTATCTGAATCATCTTTTGCCGAAACAGAAGACGGTACTTTATATTGATTCGCAAGA     2940
3           ------------------------------------------------------------     866
UF31        AACCTATCTGAATCATCTTTTGTCGAAACAGAAGACGGTACTTTATATTGATTCGCAAGA     2940
UF33        AACCTATCTGAATCATCTTTTGTCGAAACAGAAGACGGTACTTTATATTGATTCGCAAGA     2940
PG14T       AACCTATCTGAATCATCTTTTGTTGAAACAGAAGACGGTACTTTATATTGATTCGCAAGA     2940
23          ------------------------------------------------------------     867
UFG4        AACCTATCTGAATCATCTTTTGTCGAAACAGAAGACGGTACTTTATATTGATTCGCAAGA     2940
22          ------------------------------------------------------------     957
13          ------------------------------------------------------------     866
19          ------------------------------------------------------------     863
5           ------------------------------------------------------------     867
6           ------------------------------------------------------------     876

Larissa     CATACAGGTAGTTTTGGACAAAATACATTTAGAACATTTATTTCAAAAAGTACAGATGGT     3000
16          ------------------------------------------------------------     471
10          ------------------------------------------------------------     904
UFG1        CATACAGGTAGTTTTGGACAAAATACATTTAGAACATTTATTTCAAAAAGTACAGATGGT     3000
3           ------------------------------------------------------------     866
UF31        CATACAGGTAGTTTTGGACAAAATACATTTAGAACATTTATTTCAAAAAGTACAGATGGT     3000
UF33        CATACAGGCCGTTTTGGGCAAAATACATTTAGAACATTTATTTCAAAAAGTACAGATGGT     3000
PG14T       CATACAGGTAGTTTTGGACAAAATACATTTAGAACATTTATTTCAAAAAGTACAGATGGT     3000
23          ------------------------------------------------------------     867
UFG4        CATACAGGCCGTTTTGGGCAAAATACATTTAGAACATTTATTTCAAAAAGCACAGATGGT     3000
22          ------------------------------------------------------------     957
13          ------------------------------------------------------------     866
19          ------------------------------------------------------------     863
5           ------------------------------------------------------------     867
6           ------------------------------------------------------------     876

Larissa     GGTATGACTTGAAGCAGTCCTGATAATGACACATCTAGAAAAGGTAAAGATATGCAAATT     3060
16          ------------------------------------------------------------     471
10          ------------------------------------------------------------     904
UFG1        GGTATGACTTGAAGCAGTCCTGATAATGACGCATCTAGAAAAGGTAAAGATATGCAAATT     3060
3           ------------------------------------------------------------     866
UF31        GGTATGACTTGAAGCAGTCCTGATAATGACGCATCTAGAAAAGGGAAAGATATGCAAATT     3060
UF33        GGTATGACTTGAAGCAGTCCTGATAATGACGCATCTAGAAAAGGTAAAGATATGCAAATT     3060
PG14T       GGTATGACTTGAAGCAGTCCTGATAATGACACATCTAGAAAAGGTAAAGATATGCAAATT     3060
23          ------------------------------------------------------------     867
UFG4        GGTATGACTTGAAGCAGTCCTGATAATGACGCATCTAGAAAAGGTAAAGATATGCAAATT     3060
22          ------------------------------------------------------------     957
13          ------------------------------------------------------------     866
19          ------------------------------------------------------------     863
5           ------------------------------------------------------------     867
6           ------------------------------------------------------------     876
```

```
Larissa       GGTAATCCTTACGATGCAAATATTTTTTCAGGTATTGATCACTTTAGATGGAAAAATAAA      3120
16            ------------------------------------------------------------      471
10            ------------------------------------------------------------      904
UFG1          GGTAATCCTTACGATGCAAATATTTTTTCAGGTATTGATCACTTTAGATGGAAAAATAAA      3120
3             ------------------------------------------------------------      866
UF31          GGCAATCCTTACGATGGAAATATTTTTTCAGGAATCTCATATTTTAAATGAAAAAATAAA      3120
UF33          GGCAATCCTTACGATGCAAATATTTTTTCAGGTATTGATCACTTTAGATGGAAAAATAAA      3120
PG14T         GGTAATCCTTACGATGCAAATATTTTTTCAGGTATTGATCACTTTAGATGGAAAAATAAA      3120
23            ------------------------------------------------------------      867
UFG4          GGCAATCCTTACGATGCAAATATTTTTTCAGGTATTGATCACTTTAGATGGAAAAATAAA      3120
22            ------------------------------------------------------------      957
13            ------------------------------------------------------------      866
19            ------------------------------------------------------------      863
5             ------------------------------------------------------------      867
6             ------------------------------------------------------------      876

Larissa       GATTATTTTATTTTCTCTCTTTCAAAATCTGCAGTCAGAAGAAATGGTTATTTATTTATA      3180
16            ------------------------------------------------------------      471
10            ------------------------------------------------------------      904
UFG1          GATTATTTTATTTTCTCTCTTTCAAAATCTGTCGTTAGAAGAAATGGTTCTTTATTTATA      3180
3             ------------------------------------------------------------      866
UF31          GATTATTTTTTATTCTCTCTTTCAAAATCTGTAGTTAGAAGAAATGGTTCTTTATTTATA      3180
UF33          GATTATTTTATTTTCTCTCTTTCAAAATCTGTAGTTAGAAGAAATGGTTCTTTATTTATA      3180
PG14T         GATTATTTTATTTTCTCTCTTTCAAAATCTGCAGTCAGAAGAAATGGTTATTTATTTATA      3180
23            ------------------------------------------------------------      867
UFG4          GATTATTTTATTTTCTCTCTTTCAAAATCTGCAGTCAGAAGAAATGGTTCTTTATTTATG      3180
22            ------------------------------------------------------------      957
13            ------------------------------------------------------------      866
19            ------------------------------------------------------------      863
5             ------------------------------------------------------------      867
6             ------------------------------------------------------------      876

Larissa       GCAGATGCAACATTCGAGAATATTGTTGAACTATTTAGGTATGATGATAATCAAAGAGAG      3240
16            ------------------------------------------------------------      471
10            ------------------------------------------------------------      904
UFG1          GCAGATGCAACATTTGAGAATATTGTTGAACTATTTAGGTATGATGATAATCAAAGAGAG      3240
3             ------------------------------------------------------------      866
UF31          GCAGATGCAACATTTGAGAATATTGTTGAACTATTTAGGTATGATGATAATCAAAGAGAG      3240
UF33          GCAGATGCAACATTTGAGAATATTATTGAACTATTTAGGTATGATGATAATCAAAGAGAG      3240
PG14T         GCAGATGCAACATTCGAGAATATTGTTGAACTATTTAGGTATGATGATAATCAAAGAGAG      3240
23            ------------------------------------------------------------      867
UFG4          GCAGATGCAACATTTGAGAATATTGTTGAACTATTTAGGTATGATGATAATCAAAGAGAG      3240
22            ------------------------------------------------------------      957
13            ------------------------------------------------------------      866
19            ------------------------------------------------------------      863
5             ------------------------------------------------------------      867
6             ------------------------------------------------------------      876
```

```
Larissa      CATTTTGCTTATAGCTATGCGCTTGTTACTAATAAAACAGAAAACTATATAGATTTTATT      3300
16           ------------------------------------------------------------      471
10           ------------------------------------------------------------      904
UFG1         CATTTTGCTTATAGCTATGCACTTGTTACTAATAAAACAGAAAACTATATAGATTTTATT      3300
3            ------------------------------------------------------------      866
UF31         CATTTTGCTTATAGCTATGCACTTGTTACTAATAAAACAGAAAACTATATAGATTTTATT      3300
UF33         CATTTTGTTTATAGCTATGCACTTGTTACTAATAAAACAGAAAACTATATAGATTTTATT      3300
PG14T        CATTTTGCTTATAGCTATGCGCTTGTTACTAATAAAACAGAAAACTATATAGATTTTATT      3300
23           ------------------------------------------------------------      867
UFG4         CATTTTGCTTATAGCTATGCACTTGTTACTAATAAAACAGAAAACTATATAGATTTTATT      3300
22           ------------------------------------------------------------      957
13           ------------------------------------------------------------      866
19           ------------------------------------------------------------      863
5            ------------------------------------------------------------      867
6            ------------------------------------------------------------      876


Larissa      AGTATTTATGAAGCTTCTGAAAGGTTTAAAATTATAGATGGTGGATTTGATAACTCTAGA      3360
16           ------------------------------------------------------------      471
10           ------------------------------------------------------------      904
UFG1         AGTATTTATGAAGCTTCTGAAAGGTTTAAAATTTTAGATGGCGGCTTTGATAACTCTAGA      3360
3            ------------------------------------------------------------      866
UF31         AGTATTTATGAAGCTTCTGAAAGGTTTAAAATTATAGATGGCGGATTTGATAATTCTAGA      3360
UF33         AGTATTTATGAAGCTTCTGAAAGGTTTAAAATTATAGATGGCGGATTTGATAATTCTAGA      3360
PG14T        AGTATTTATGAAGCTTCTGAAAGGTTTAAAATTATAGATGGTGGATTTGATAACTCTAGA      3360
23           ------------------------------------------------------------      867
UFG4         AGTATTTATGAAGCTTCTGAAAGGTTTAAAATTTTAGATGGCGGCTTTGATAACTCTAGA      3360
22           ------------------------------------------------------------      957
13           ------------------------------------------------------------      866
19           ------------------------------------------------------------      863
5            ------------------------------------------------------------      867
6            ------------------------------------------------------------      876


Larissa      CCAAAAGGTGGAGAAATCCAATTAGACAAATTTAGATTATGAATTAAAGATTAA    3414
16           ------------------------------------------------------    471
10           ------------------------------------------------------    904
UFG1         CCACAAGGTGGAGAAATTCAGTTAGATAAATTTAGATTATGAATTAAAGATTAA    3414
3            ------------------------------------------------------    866
UF31         CCACAAGGTGGAGAAATTCAGTTAGATAAATTTAGATTATGAATTAAAGATTAA    3414
UF33         CCACAAGGTGGAGAAATTCAGTTAGATAAATTTAGATTATGAATTAAAGATTAA    3414
PG14T        CCAAAAGGTGGAGAAATCCAATTAGACAAATTTAGATTATGAATTAAAGATTAA    3414
23           ------------------------------------------------------    867
UFG4         CCACAAGGTGGAGAAATTCAGTTAGATAAATTTAGATTATGAATTAAAGATTAA    3414
22           ------------------------------------------------------    957
13           ------------------------------------------------------    866
19           ------------------------------------------------------    863
5            ------------------------------------------------------    867
6            ------------------------------------------------------    876
```

The neuraminidase partial nucleotide sequences are also available in GenBank under accession numbers PP430513 (patient 3), PP430514 (patient 5), PP430515 (patient 6), PP430516 (patient 10), PP430517 (patient 13), PP430518 (patient 16), PP430519 (patient 19), PP430520 (patient 22), PP430521 (patient 23).
