# Supplementary material for: Association of Mycoplasma canis with Fertility Disorders in Dogs: A Case Study Supported by Clinical Examination, PCR, 16S Microbiota Profiling, and Serology
Source: Pathogens. 2024 May 8;13(5):391. doi: 10.3390/pathogens13050391 (PMC11123722; doi:10.3390/pathogens13050391)
Supplement: Supplementary file 1 [file pathogens-13-00391-s001.zip › Table S1.pdf]

Table S1. Representation of all numerical values of monitored hematological and biochemical parameters for patients in FD and CTRL group. FD, fertility disorder; CTRL, control.

Study group	Patient number	Breed	Sex	WBC [×10^9/L]	RBC [×10^{12}/L]	HGB [g/L]	HCT [L/L]	MCV [fL]	MCH [pg]	MCHC [g/L]	CHCM [g/L]	CH [pg]	RDW [%]	HDW [g/L]	PLT [×10^9/L]	MPV [fL]	PCT [%]	%NEUT [%]	%LYMPH [%]	%MONO [%]	%EOS [%]	%BASO [%]	%LUC [%]	#NEUT [×10^9/L]	#LYMPH [×10^9/L]	#MONO [×10^9/L]	#EOS [×10^9/L]	#BASO [×10^9/L]	#LUC [×10^9/L]	urea [mmol/L]	creatinine [µmol/L]	total proteins [mg/mL]	IgG [mg/mL]
FD	1	Parson Russel terrier	M	13.77	7.97	185	0.53	66.6	23.2	348	343	22.8	12.6	17.6	262	8.6	0.23	68.4	20.7	4.5	6.2	0.2	0.1	9.41	2.84	0.62	0.85	0.03	0.01	6.60	67.50	54.95	37.46
	2	Bernese mountain dog	M	12.80	7.63	185	0.54	70.6	24.3	344	326	23.0	11.5	15.6	163	14.2	0.23	65.1	23.2	4.5	6.9	0.2	0.1	7.93	2.82	0.55	0.84	0.02	0.02	9.20	101.85	57.57	41.59
	3	German shepherd	F	6.80	8.55	195	0.57	66.9	22.8	340	348	23.2	12.5	18.3	159	13.7	0.22	54.3	32.1	6.4	6.6	0.3	0.3	3.69	2.18	0.44	0.45	0.02	0.02	5.65	78.74	ND	ND
	4	German shepherd	F	7.28	7.98	184	0.55	69.0	23.0	333	354	24.4	11.8	15.7	264	10.4	0.27	50.4	24.6	4.8	19.7	0.2	0.2	3.67	1.79	0.35	1.44	0.02	0.01	5.33	63.43	55.12	41.36
	5	Golden retriever	F	10.75	7.20	170	0.47	65.9	23.5	357	369	24.3	13.7	18.8	286	9.0	0.26	64.0	27.5	3.3	4.9	0.1	0.1	6.88	2.96	0.36	0.53	0.01	0.01	5.74	102.31	57.65	36.95
	6	Golden retriever	M	14.09	6.45	154	0.44	68.8	23.8	346	366	25.2	12.9	16.9	219	10.3	0.23	64.3	27.1	3.7	4.6	0.1	0.2	9.05	3.82	0.52	0.65	0.01	0.03	7.21	137.00	58.04	37.50
	7	Airedale terrier	F	6.12	8.44	193	0.55	65.0	22.9	352	365	23.7	12.7	20.1	198	8.8	0.17	63.9	25.9	3.9	6.0	0.3	0.1	3.91	1.59	0.24	0.36	0.02	0.01	12.23	89.00	54.33	40.56
	8	Airedale terrier	M	9.40	7.60	178	0.49	64.0	23.4	365	371	23.8	14.5	21.4	292	9.4	0.28	60.1	29.0	3.6	6.9	0.2	0.1	5.65	2.72	0.34	0.65	0.02	0.01	14.73	85.00	56.99	41.16
	9	German shepherd	F	12.17	6.57	148	0.42	63.2	22.5	356	371	23.4	13.0	18.2	101	14.7	0.15	62.6	24.7	6.0	6.2	0.1	0.3	7.62	3.01	0.74	0.75	0.02	0.03	5.88	98.00	56.66	32.62
	10	American Staffordshire terrier	F	10.11	7.13	165	0.47	65.3	23.1	354	356	23.2	13.5	20.5	412	11.9	0.49	73.3	15.6	6.1	3.8	0.5	0.7	7.41	1.58	0.62	0.39	0.05	0.02	4.81	92.20	56.33	42.25
	11	Dogo Argentino	F	10.49	2.40	57	0.17	71.3	23.9	335	356	25.3	14.0	20.2	180	13.9	0.25	71.9	18.4	6.6	2.7	0.2	0.3	7.54	1.93	0.69	0.28	0.02	0.03	3.06	61.42	31.53	24.02
	12	Bernese mountain dog	F	10.85	8.03	185	0.53	65.8	23.1	351	361	23.7	13.1	18.6	297	11.6	0.35	74.1	19.1	5.3	1.1	0.1	0.1	8.04	2.07	0.58	0.12	0.01	0.01	6.49	109.06	57.69	41.56
	13	Bernese mountain dog	F	15.71	7.29	168	0.46	63.8	23.1	362	355	22.6	13.7	20.5	400	13.8	0.55	77.4	14.1	4.8	3.3	0.1	0.1	12.7	2.22	0.76	0.52	0.02	0.02	5.96	150.10	56.48	42.47
	14	Weimaraner	F	8.24	6.52	160	0.46	70.8	24.5	347	347	24.5	12.3	17.2	326	10.2	0.33	77.6	14.5	4.8	2.9	0.2	0.1	6.39	1.20	0.39	0.23	0.02	0.01	4.32	80.03	48.33	38.26
CTRL	15	English springer spaniel	F	7.82	6.84	163	0.50	72.2	23.8	328	322	23.4	14.4	16.7	147	16.5	0.24	70.3	17.1	5.7	6.4	0.2	0.3	5.50	1.34	0.45	0.50	0.02	0.03	6.36	96.47	55.02	39.14
	16	Cross breed	F	9.61	7.25	175	0.52	72.1	24.1	334	328	23.6	11.6	14.7	290	9.2	0.27	50.2	31.9	3.6	13.8	0.3	0.1	4.83	3.06	0.35	1.32	0.03	0.01	10.63	101.12	53.87	41.49
	17	Japanese chin	M	8.11	6.32	148	0.45	70.4	23.4	332	321	22.6	11.8	15.3	289	9.2	0.27	67.4	24.1	4.3	3.9	0.2	0.2	5.47	1.95	0.35	0.31	0.01	0.01	6.64	129.75	55.90	36.67
	19	Scottish terrier	M	7.21	7.71	163	0.48	62.7	21.2	338	326	20.4	13.6	17.8	293	11.0	0.32	54.3	32.1	7.4	5.1	0.7	0.4	3.92	2.32	0.53	0.37	0.05	0.03	3.08	65.35	55.49	40.98
	20	Scottish terrier	M	14.40	8.21	181	0.54	65.8	22.1	336	325	21.3	12.9	17.3	234	9.6	0.22	69.7	21.6	3.9	4.1	0.5	0.3	10.04	3.11	0.56	0.58	0.07	0.04	5.03	79.22	58.74	42.72
	21	Rottweiler	M	14.35	6.48	142	0.42	65.0	21.9	337	323	21.0	12.2	17.9	481	9.3	0.45	45.2	24.6	3.3	25.9	0.2	0.9	6.49	3.53	0.47	3.71	0.02	0.13	4.52	87.20	55.89	41.28
	22	Rottweiler	M	15.60	7.70	174	0.50	65.4	22.7	346	335	21.8	11.8	16.7	311	9.8	0.31	46.7	30.0	4.0	18.8	0.3	0.3	7.28	4.69	0.62	2.93	0.04	0.04	7.16	113.76	56.81	41.98
	23	Dobermann	M	18.50	5.82	132	0.38	64.7	22.7	352	358	23.1	12.1	20.5	253	11.4	0.29	78.4	9.3	7.8	4.4	0.0	0.1	14.50	1.73	1.45	0.81	0.00	0.01	22.70	312.80	56.73	41.57
	29	Borzoi	M	11.04	7.82	190	0.55	70.9	24.3	343	356	25.2	12.3	17.2	194	11.0	0.21	58.5	29.8	2.8	8.4	0.2	0.2	6.46	3.30	0.31	0.93	0.02	0.02	5.87	11.30	53.49	42.16
	32	Cross breed	M	9.15	5.60	128	0.38	68.2	22.9	336	350	23.8	13.5	23.6	402	12.8	0.52	69.7	18.1	4.8	7.1	0.1	0.1	6.37	1.66	0.44	0.65	0.01	0.01	5.33	57.57	57.50	41.58
	33	Borzoi	M	7.40	6.20	150	0.44	70.2	24.1	344	356	24.9	13.1	16.8	216	10.3	0.22	55.4	32.2	5.2	6.5	0.4	0.3	4.10	2.39	0.38	0.48	0.03	0.02	6.56	89.94	50.58	36.87
	40	Golden retriever	M	13.93	6.78	162	0.47	68.8	24.0	348	355	24.4	12.8	19.9	236	9.2	0.22	59.5	33.7	4.5	1.6	0.4	0.3	8.29	4.69	0.63	0.22	0.06	0.04	10.30	87.00	54.09	41.51
	41	Golden retriever	F	9.00	7.41	178	0.51	68.6	24.0	349	360	24.7	12.4	16.0	460	7.5	0.35	65.5	22.9	5.1	6.0	0.2	0.2	5.90	2.06	0.46	0.54	0.02	0.01	11.56	77.00	56.79	37.72
	42	Cross breed	F	12.84	8.59	202	0.58	67.7	23.6	348	370	25.0	12.9	18.2	499	10.1	0.50	62.6	20.4	3.7	12.6	0.5	0.1	8.04	2.62	0.48	1.62	0.07	0.02	4.13	70.50	54.85	40.49
	43	Maltese dog	M	12.71	5.62	138	0.38	67.0	24.6	367	361	24.2	12.4	20.4	538	9.3	0.50	67.2	22.0	4.9	5.4	0.1	0.3	8.54	2.80	0.62	0.69	0.01	0.04	9.47	84.80	50.65	38.48
	44	Weimaraner	M	10.87	7.77	183	0.51	65.9	23.5	357	365	24.0	12.9	18.1	446	10.7	0.48	64.2	14.9	4.0	16.7	0.2	0.2	6.98	1.62	0.44	1.81	0.02	0.02	8.95	76.13	57.12	42.20
