# Supplementary material for: Association of Mycoplasma canis with Fertility Disorders in Dogs: A Case Study Supported by Clinical Examination, PCR, 16S Microbiota Profiling, and Serology
Source: Pathogens. 2024 May 8;13(5):391. doi: 10.3390/pathogens13050391 (PMC11123722; doi:10.3390/pathogens13050391)
Supplement: Supplementary file 1 [file pathogens-13-00391-s001.zip › Table S2.pdf]

Table S2. Sperm parameters of four males from CTRL group.

Patient number	Examination of the semen immediately after collection					Microscopic examination of the semen				Eosin-nigrosin test [%]		CASA analysis											
	Colour	Odour	Sperm count/mL	Sperm volume [mL]	Sperm mobility [%]	MNS [%]	MAS [%]	MNS [%] after 3 hours	MAS [%] after 3 hours	at the time of collection	after 3 hours	% TM 0 h (1st measurement)	% TM 0 h (2nd measurement)	% TM 0 h (average)	% PM 0h (1st measurement)	% PM 0h (2nd measurement)	% PM 0h (average)	% TM 3 h (1st measurement)	% TM 3 h (2nd measurement)	% TM 3 h (average)	% PM 3 h (1st measurement)	% PM 3 h (2nd measurement)	% PM 3 h (average)
19	Milky white	None	$35.4×10^6$	3.4	85	78	22	48	52	56	88	19	43/39	41	8	20/14	17	24	29	27	7	10	9
20	Milky white	None	$67.3×10^6$	5.2	90	84	16	64	36	64	73	87	81	84	60	55	58	89	89	89	45	44/49	46
21	Milky white	None	$41.5×10^6$	2.5	90	51	49	42	58	58	66	16	19	18	0	1	1	19	18	19	2	0	1
22	Milky white	None	$37.4×10^6$	6.0	90	76	24	79	21	51	82	93	92/92	92	72	64/62	63	92	89	91	70	66	68

MNS - morphologically normal sperm, MAS - morphologically abnormal sperm, TM – total motility, PM – progressive motility
