# Supplementary material for: Association of Mycoplasma canis with Fertility Disorders in Dogs: A Case Study Supported by Clinical Examination, PCR, 16S Microbiota Profiling, and Serology
Source: Pathogens. 2024 May 8;13(5):391. doi: 10.3390/pathogens13050391 (PMC11123722; doi:10.3390/pathogens13050391)
Supplement: Supplementary file 1 [file pathogens-13-00391-s001.zip › Table S3.pdf]

Table S3. Results of 16S microbiota profiling. Results in % represent bacterial species composition of each sample. FD, fertility disorder; CTRL, control.

**Study group**	**FD**					**CTRL**				
**Patient number**	**1**	**6**	**8**	**9**	**11**	**16**	**17**	**23**	**42**	**43**
**Species**										
Mycoplasma canis	0.00	2.48	0.00	0.00	0.00	10.77	0.01	0.23	0.00	0.24
Mycoplasma cynos	11.76	0.00	17.49	0.00	0.00	0.09	18.50	66.46	0.00	90.85
Mycoplasma opalescens	2.10	0.00	0.10	0.00	0.00	0.00	0.09	0.28	0.00	0.30
Mycoplasma fermentans	0.02	0.00	0.00	0.00	0.00	0.00	0.00	0.00	0.00	0.00
Mycoplasma spumans	0.00	0.00	0.00	0.00	0.00	0.00	0.00	0.38	0.00	0.00
Mycoplasma arginini	0.00	0.00	0.00	0.00	0.00	0.00	0.00	0.04	0.00	0.00
Mycoplasma canadense	0.00	0.00	0.00	0.00	0.00	0.00	0.00	0.02	0.00	0.00
Ureaplasma canigenitalium	81.37	0.00	72.39	0.00	0.23	0.00	75.90	32.47	0.00	0.00
Haemophilus haemoglobinophilus	4.19	0.00	9.51	68.77	31.84	0.44	4.88	0.00	0.0	6.62
Haemophilus felis	0.00	0.00	0.00	0.36	0.00	0.00	0.07	0.00	0.00	0.00
Haemophilus ducreyi	0.01	0.00	0.00	0.00	0.01	0.00	0.00	0.00	0.00	0.00
Streptococcus canis	0.00	0.00	0.00	0.00	1.64	56.36	0.00	0.00	0.00	1.07
Streptococcus agalactiae	0.00	0.00	0.00	0.00	0.00	0.54	0.00	0.00	0.00	0.00
Streptococcus equi	0.00	0.00	0.00	0.00	0.31	0.48	0.00	0.00	0.29	0.00
Streptococcus dysgalactiae	0.00	0.00	0.00	0.00	0.00	1.31	0.00	0.00	0.12	0.00
Streptococcus pyogenes	0.00	0.00	0.00	0.00	0.00	1.14	0.00	0.00	0.00	0.00
Streptococcus urinalis	0.00	0.00	0.00	0.00	0.54	0.00	0.00	0.00	0.00	0.00
Streptococcus castoreus	0.00	0.00	0.00	0.00	0.00	0.41	0.00	0.00	0.00	0.00
Streptococcus didelphis	0.00	0.00	0.00	0.00	0.00	0.13	0.00	0.00	0.00	0.00
Streptococcus intermedius	0.00	0.00	0.00	0.00	0.00	0.00	0.00	0.00	0.27	0.00
Streptococcus caballi	0.00	0.00	0.00	0.00	0.00	0.00	0.00	0.00	0.26	0.00
Fusobacterium sp. HMSC073F01	0.00	0.00	0.00	0.00	0.45	0.00	0.00	0.00	0.00	0.00
Fusobacterium gonidiaformans	0.00	0.00	0.00	0.00	0.00	0.00	0.00	0.00	0.81	0.00
Fusobacterium varium	0.00	0.00	0.00	0.00	0.38	0.00	0.00	0.00	0.00	0.00
Fusobacterium necrophorum	0.00	0.00	0.00	0.00	0.00	0.00	0.00	0.00	87.21	0.00
Fusobacterium russii	0.00	0.00	0.00	0.00	0.39	0.00	0.00	0.00	0.03	0.00
Psychrobacter sp. SHUES1	0.00	61.09	0.00	0.00	0.00	0.00	0.00	0.00	0.00	0.00
Psychrobacter sp. G	0.00	4.19	0.00	0.00	0.00	0.00	0.00	0.00	0.00	0.00
Psychrobacter cryohalolentis	0.00	4.18	0.00	0.00	0.00	0.00	0.00	0.00	0.00	0.00
Psychrobacter sp. 1501(2011)	0.00	3.80	0.00	0.00	0.00	0.00	0.00	0.00	0.00	0.00
Psychrobacter sp. P11F6	0.00	0.92	0.00	0.00	0.00	0.00	0.00	0.00	0.00	0.00
Psychrobacter alimentarius	0.00	2.77	0.00	0.00	0.00	0.00	0.00	0.00	0.00	0.00
Psychrobacter glacincola	0.00	0.98	0.00	0.00	0.00	0.00	0.00	0.00	0.00	0.00
Porphyromonas gingivalis	0.00	0.00	0.00	0.26	1.10	0.00	0.00	0.00	0.31	0.00
Porphyromonas cangingivalis	0.00	0.00	0.00	19.58	11.37	0.00	0.00	0.00	0.05	0.00
Porphyromonas gingivicanis	0.00	0.00	0.00	5.08	0.43	0.00	0.00	0.00	0.09	0.00
Porphyromonas crevioricanis	0.00	0.00	0.00	0.69	0.20	0.00	0.14	0.00	0.55	0.00
Porphyromonas gulae	0.00	0.00	0.00	0.80	0.68	0.00	0.00	0.00	0.16	0.00
Porphyromonas circumdentaria	0.00	0.00	0.00	1.09	0.18	0.00	0.00	0.00	0.19	0.00
Bacteroides pyogene	0.00	0.00	0.00	0.00	24.34	0.00	0.00	0.00	0.00	0.00
Bacteroides fragilis	0.00	0.00	0.00	0.00	2.73	0.00	0.00	0.00	0.02	0.00
Pseudomonas fragi	0.00	2.09	0.00	0.00	0.00	0.00	0.00	0.00	0.00	0.00
Staphylococcus equorum	0.00	0.62	0.00	0.00	0.00	0.00	0.00	0.00	0.00	0.00
Pasteurella multocida	0.00	0.00	0.00	0.18	0.00	0.00	0.00	0.00	0.00	0.00
Escherichia coli	0.00	0.00	0.00	0.00	1.61	3.83	0.00	0.00	0.00	0.00
Histophilus somni	0.00	0.00	0.08	0.27	0.49	0.00	0.08	0.00	0.00	0.15
Lactobacillus gasseri	0.00	0.00	0.00	0.00	0.00	0.00	0.00	0.00	0.00	0.01
Prevotella intermedis	0.00	0.00	0.00	0.00	0.12	0.00	0.00	0.00	0.25	0.00
Weissella confusa	0.00	0.00	0.00	0.00	0.00	2.23	0.00	0.00	0.00	0.00
Nostoc linckia	0.01	0.01	0.03	0.08	0.00	0.01	0.01	0.00	0.00	0.04
Peptoniphilus indolicus	0.00	0.00	0.00	0.00	0.27	0.00	0.00	0.00	0.19	0.00
Moraxella atlantae	0.00	0.00	0.00	0.00	0.00	0.00	0.00	0.00	0.00	0.01
Actinobacillus suis	0.01	0.00	0.00	0.00	0.00	0.00	0.00	0.00	0.00	0.00
Aerococcus christensenii	0.00	0.00	0.00	0.00	0.00	0.00	0.00	0.01	0.00	0.00
Other	0.55	11.93	0.40	2.61	18.89	19.51	0.30	0.11	6.40	0.72
