# Supplementary material for: Association of Mycoplasma canis with Fertility Disorders in Dogs: A Case Study Supported by Clinical Examination, PCR, 16S Microbiota Profiling, and Serology
Source: Pathogens. 2024 May 8;13(5):391. doi: 10.3390/pathogens13050391 (PMC11123722; doi:10.3390/pathogens13050391)
Supplement: Supplementary file 1 [file pathogens-13-00391-s001.zip › Table S4.pdf]

Table S4. Alpha diversity metrics of the 10 samples that were included in the final 16S microbiota profiling. FD, fertility disorder; CTRL, control; M, male; F, female.

Study group	Patient number	Sex	Sample number[a]	Chao1 index	Shannon index	Simpson index	Observed OTUs
FD	1	M	6SI	17.0000	0.9830	0.3270	14
	6	M	38SI	197.7692	2.6827	0.6151	125
	8	M	43SI	14.7500	1.1632	0.4367	14
	9	F	44SI	47.0000	1.7829	0.5020	45
	11	F	49SI	175.1538	3.9402	0.8543	137
CTRL	16	F	3SI	160.6667	2.8504	0.6667	121
	17	M	4SI	34.0000	1.0280	0.3810	22
	23	M	90SI	12.0000	1.0236	0.4542	12
	42	F	45SI	200.6667	2.2602	0.5281	138
	43	M	47SI	24.2000	0.5928	0.1692	23

[a] in Figures 1 and 2 individual patients are represented with sample number (as assigned by Diversigen Inc.) instead of patient number
